# MoS₂-DNA tetrahedral bioconjugate for high-performance DNA biosensors: application in viral infection diagnostics

**DOI:** 10.1007/s00604-025-07084-2

**Published:** 2025-03-11

**Authors:** Estefanía Enebral-Romero, Emiliano Martínez-Periñán, David López-Diego, Mónica Luna, Marina Garrido, Cristina Navío, Emilio M. Pérez, Encarnación Lorenzo, Tania García-Mendiola

**Affiliations:** 1 IMDEA-Nanociencia, Ciudad Universitaria de Cantoblanco, 28049 Madrid, Spain; 2 Departamento de Química Analítica y Análisis Instrumental, Universidad Autónoma de Madrid, 28049 Madrid, Spain; 3 Institute for Advanced Research in Chemical Sciences (IAdChem), Universidad Autónoma de Madrid, 28049 Madrid, Spain; 4 Instituto de Micro y Nanotecnología IMN-CNM, CSIC (CEI UAM+CSIC), Tres Cantos, Isaac Newton 8, 28760 Madrid, Spain

**Keywords:** Bioconjugate, Biosensor, Differential pulse voltammetry, Molybdenum disulphide, Tetrahedral DNA nanostructures, Thionine-modified carbon nanodots

## Abstract

An electrochemical DNA biosensor is presented for early viral infection detection, integrating molybdenum disulphide (MoS₂), tetrahedral DNA nanostructures (TDNs), and thionine-modified carbon nanodots (CNDsTy). The innovation of this work lies in the first-time integration of these nanomaterials for the preparation of a bioconjugate, whose synergy enables the biosensor’s functionality. MoS₂ anchors the TDNs, which carry the capture probe for virus identification via genetic code recognition. CNDsTy allow the electrochemical detection based on their different affinity for single-stranded (ssDNA) and double-stranded DNA (dsDNA), enabling hybridization event identification. The biosensor achieves high sensitivity (detection limit of 5.00 fM) and can distinguish viral loads, validated with the SARS-CoV-2 ORF1ab sequence in human nasopharyngeal samples.

**Graphical Abstract:**

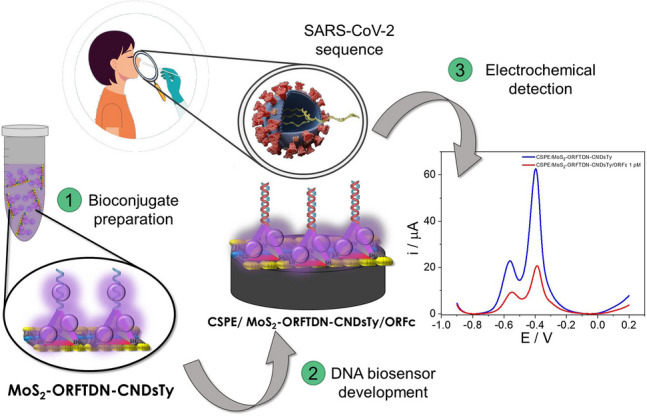

**Supplementary Information:**

The online version contains supplementary material available at 10.1007/s00604-025-07084-2.

## Introduction

MoS_2_, a thermodynamic stable semiconducting transition metal dichalcogenide (TMDC), has attracted the attention of the scientific community in recent years as a graphene-like material with excellent properties that allow its application in a wide variety of fields [1, 2]. Thanks to its surface-to-volume ratio, weak interplanar Van der Waals interactions, and its reduced layer thickness, MoS_2_ has a high relative surface area with the atoms mainly located on the surface, which translates into a multitude of active sites and a great capacity for functionalization, allowing the adsorption of biomolecules on its surface [3]. Its tuneable band gap and optical transparency give this 2D nanomaterial excellent electrical and optoelectronic properties, with high electrical conductivity and electron mobility, excellent photoluminescent properties, good catalytic properties, as well as low toxicity and high biocompatibility, among others [4]. Therefore, MoS_2_ has been used in the development of energy storage devices, in catalysis, in optoelectronic applications, for the development of photodetectors or light-emitting devices, or in photovoltaic applications [5, 6]. Meanwhile, this nanomaterial is in the early stages of its application in the development of electrochemical sensors and biosensors. Compared to other conventional 2D nanomaterials, MoS_2_ is an excellent candidate for the development of electrochemical biosensors, favouring the immobilization of different biomolecules on the surface of the biosensing platform and improving the performance of the device.

MoS_2_ can be combined with other 2D or 3D nanomaterials or nanostructures and biomolecules, giving rise to nano-heterostructures such as bioconjugates, with better electrical properties [7]. The use of these bioconjugates in biosensors enables the development of simpler and faster platforms with enhanced analytical properties in terms of sensitivity, specificity, and stability [8]. These advancements stem from the synergistic properties provided by the integration of 2D nanomaterials with biomolecules or nanostructures, creating a more effective methodology to detection and analysis.

Over the years, new methodologies for immobilizing DNA probes have been developed in biosensor technology, aiming to enhance sensitivity and specificity to construct new biosensing devices that can meet the requirement for commercial use [9]. In this sense, DNA nanostructures have emerged as an innovative approach, primarily involving the preparation of 3D polyhedral nanostructures through the nitrogenous bases complementarity, such as tetrahedral DNA nanostructures (TDN), which exhibit high stability, rigidity, high reaction yields, and a high capacity for functionalization with different biomolecules or other functional groups [10]. This allows the design of specific TDNs for the detection of different pathogens, functionalizing the top vertex of the TDN with the specific capture probe, and the immobilization of the same on the electrode surface, modifying the basal vertices with functional groups such as the amino or thiol group (–NH_2_ or –SH respectively) [11, 12]. There is a wide range of research that demonstrates the high versatility that TDNs offer for the detection of all types of analytes of great interest (biomolecules and other molecules), from the detection of viruses and pathogens from DNA sequences [13], the detection of biomarkers associated with different diseases such as cancer from miRNAs [14, 15], protein detection, from aptamers as a capture probe [11], or detection of metal ions among others [16], and which reflect the wide spectrum of applications that these nanostructures can cover, such as biosensing and bioimaging, drug delivery, or in nanomedicine [17–19]. Furthermore, combining these DNA nanostructures with 2D nanomaterials, such as MoS_2_, enables the preparation of novel bioconjugates, due to the interaction between the thiol groups of the TDNs and the sulphur vacances in the nanomaterial [20]. These bioconjugates can be directly immobilized onto electrode surfaces, reducing the steps in biosensors development. Moreover, as the TDNs have a pyramidal shape with three anchor points, the immobilization on the 2D nanomaterial will be stronger and more stable, and the capture probes will be more spaced and vertically oriented, thus favouring the hybridization with the analyte sequence and subsequently improving the analyte detection [19].

In the development of electrochemical DNA biosensors one of the most attractive strategies for the detection of the hybridization event due its simplicity and low cost is the use of electrochemical indicators. Some of the most used electrochemically active molecules are phenothiazines, such as thionine. Although thionine presents excellent redox properties and is a good candidate for the electrochemical detection of the hybridization event, recent studies propose the use of functionalized carbon nanodots (CDs) with phenotiazines, as new potential electrochemical indicators to improve analytical properties of DNA biosensors and develop new devices that can overcome the current challenges of the biosensor technologies [21, 22]. In particular, we have recently reported the potential applicability of CDs modified with thionine as electrochemical indicators since they present different affinity for the single-stranded DNA (ssDNA) or the double-stranded DNA (dsDNA), allowing the detection of the hybridization event [23].

Based on the above, this work proposes the preparation of a bioconjugate based on the combination of MoS_2_, TDNs, and thionine-modified carbon nanodots (CDsTy). The MoS_2_ nanoflakes act as anchoring points for the thiolated TDNs, which carry the capture probe for the virus detection by its genetic code. The bioconjugate has been redox labelled with CNDsTy that act as electrochemical indicators of the hybridization event. The proposed bioconjugate has been applied for the development of a rapid, simple, low-cost, portable, miniaturized, and sensitive electrochemical biosensor for the detection of the SARS-CoV-2 specific ORF1ab sequence as a model of virus detection.

## Experimental section

### Methods

#### Liquid-phase exfoliation (LPE) of molybdenum disulphide flakes

The MoS_2_ flakes used in this work have been prepared by LPE, using a methodology previously published [24]. Briefly, 200 mg of bulk MoS_2_ was dispersed in 200 mL of *N*-methyl-2-pyrrolidone (NMP). The mixture was sonicated during 1 h by an ultrasonic probe, at 35% of amplitude, under an ice bath to avoid the increase of temperature. The obtained black suspension was centrifuged for 30 min at 5000 rpm yielding a black sediment (MoS_2_ non-exfoliated) and an olive-colour supernatant where the exfoliated MoS_2_ flakes were located. Finally, this supernatant was filtered using PTFE membranes filters (0.45 µm) and washed by redispersion and filtration three times in acetonitrile and three times in isopropanol, obtaining the exfoliated MoS_2_.

#### Thionine functionalized carbon nanodots (CNDsTy) synthesis

CNDsTy were synthesized following the procedure previously published by the research group [23]. The mixture of L-arginine, 3,3′-diamino-N-methyldipropylamine, thionine acetate salt, and Milli-Q water was irradiated in a microwave system at 235 °C and 20 bar for 180 s. The blue solid prepared was dissolved in ultra-pure water and filtered with a 0.1 µm porous filter, and the CNDsTy were finally obtained.

#### Tetrahedral DNA nanostructure synthesis

The process followed for the preparation of the tetrahedral DNA nanostructure used in this work has been previously optimized by the research group [13]. Briefly, for 1.00 µM TDN synthesis, equimolar amounts of four oligonucleotides (ORF-Tetra A, Tetra-B, Tetra-C, and Tetra-D) were mixed with TM buffer (20.00 mM Tris, 50.00 mM MgCl_2_ pH 8.0). Then, the mixture was subjected into a thermocycler to a thermostatized process of three stages divided in two different steps of 2 min each. First, temperatures of 95 °C and 51 °C were applied. Then, in the second stage, decreasing temperatures of 46.1 °C, 43.6 °C, 41.2 °C, and 38.8 °C were employed. Finally, two steps of 30 °C and 4 °C were done.

#### Bioconjugate (MoS2-ORFTDN-CNDsTy) preparation

To prepare the MoS_2_-ORFTDN-CNDsTy bioconjugate, 100 µL of the ORFTDN (1.00 µM) synthesized and 100 µL of the CNDsTy (2.83 mg/mL) were mixed and left in the dark for 1 h. The mixture was then filtered with a 3 K filter in a centrifuge during 10 min at 10,000 rpm to separate the unbound molecules. Next, sterilized Milli-Q water was added to the filter, and the same process was carried out until the filter volume was halved, to wash the prepared bioconjugate solution. Finally, 100 µL of MoS_2_ and 100 µL of the ORFTDN-CNDsTy complex were mixed overnight in dark and then filtered during 3 min at 7000 rpm, obtaining the MoS_2_-ORFTDN-CNDsTy bioconjugate desired.

#### Biosensor development and SARS-CoV-2 detection

Firstly, a Carbon Screen-Printed electrode (CSPE) was modified by drop casting with 10.0 µL of the bioconjugate (CSPE/MoS_2_-ORFTDN-CNDsTy) and left overnight in dark conditions. The day after, the electrode was washed with sterilized water. For SARS-CoV-2 virus detection, the biosensing platform developed was then incubated with 10.0 µL of the ORF1ab specific sequence at different concentrations, for an hour at 40 °C in a humidity chamber under stirring and washed with Milli-Q water. Finally, the electrochemical detection of the virus was achieved by Differential Pulse Voltammetry (DPV) in an electrolyte solution of PB 0.1 M pH 7.0.

#### SARS-CoV-2 virus detection in nasopharyngeal human samples

For the validation of the developed biosensor, inactivated nasopharyngeal samples containing SARS-CoV-2 viral RNA, which were provided by the “Instituto Ramón y Cajal de Investigación Sanitaria” (IRYCIS) of the Autonomous Community of Madrid, were analyzed. RNA was extracted from the swab samples using the QIAamp Viral RNA Qiagen kit, it was eluted in RNase-free water, its concentration was measured using a Nanodrop, and it was stored at − 80 °C. All processes were carried out in P2 biosafety hoods, following recommendations and protocols to avoid sample degradation or possible cross-contamination between negative and positive samples. Samples from one non-infected patient and two infected patients with different viral loads, high and low (with Ct values of 12 and 30, respectively), were analyzed by RT-qPCR. These same samples were studied with the developed biosensor. For that, the CSPE/MoS_2_-ORFTDN-CNDsTy platform was incubated with 10.0 µL of the nasopharyngeal human samples. After incubation (1 h, 40 °C, under stirring), electrodes were washed with purified water, and electrochemical measurements were performed using PB 0.1 M pH 7.0 as electrolyte. From the DPVs registered, intensity values of each sample were recorded and normalized with the blank. Results were compared to evaluate the ability of the biosensor to detect the virus, and to differentiate between different viral loads.

## Results and discussion

**Scheme 1:**
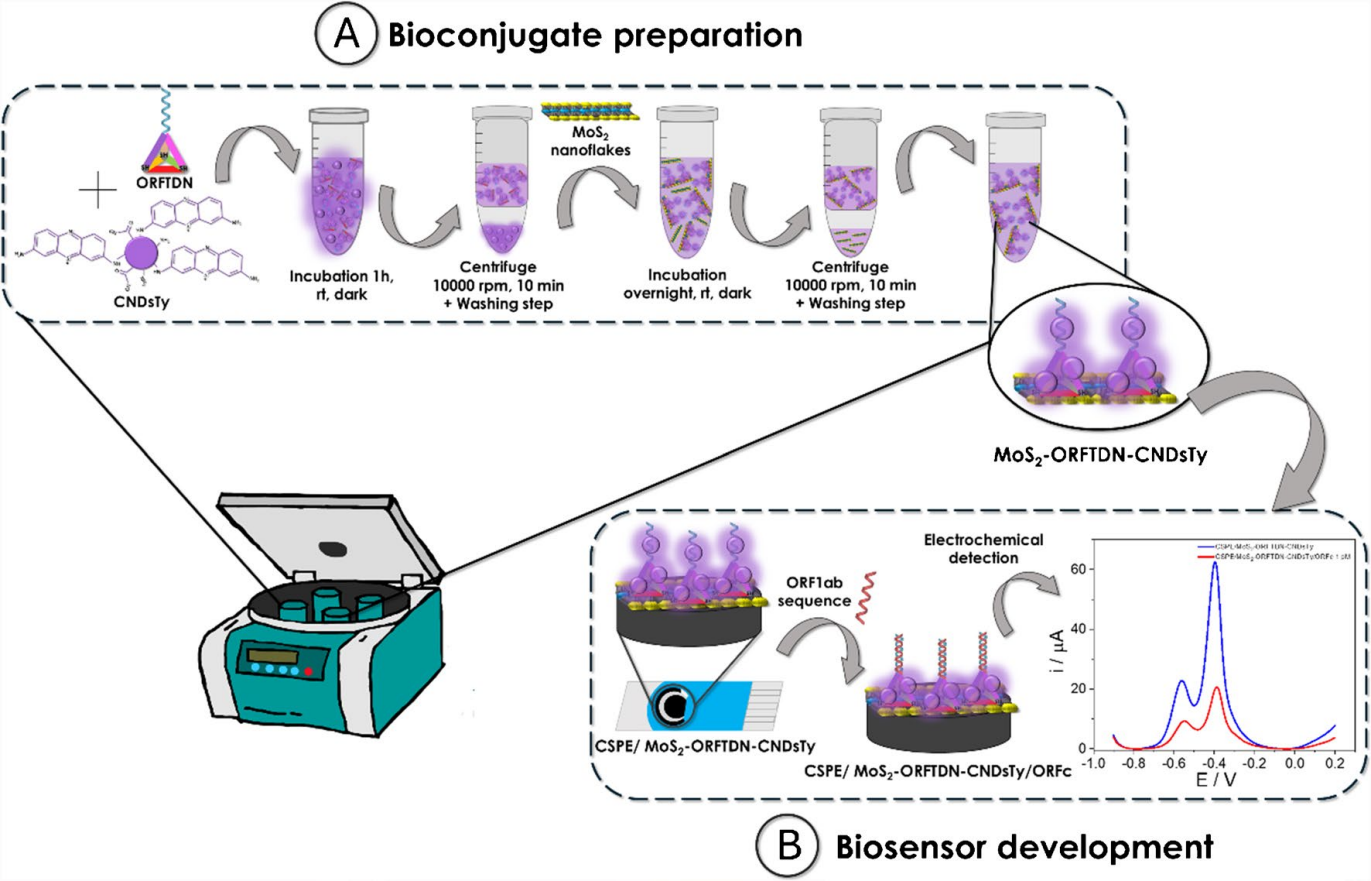
Scheme followed for the bioconjugate preparation (**A**) and the electrochemical DNA biosensor development (**B**)

Scheme 1 shows the process followed for the bioconjugate preparation and the electrochemical DNA biosensor development proposed in this work. Firstly, the bioconjugate is prepared by incubation of the synthesized tetrahedral DNA nanostructures, containing the probe sequence (ORFTDN) as biorecognition element, with the thionine-modified carbon nanodots (CNDsTy) as redox indicator. Then ORFTDN-CNDsTy are immobilized on the molybdenum disulphide nanoflakes (MoS_2_) taking advantage of the interactions between the sulphur vacances from the MoS_2_ nanoflakes and the thiol groups of the ORFTDN. Finally, the bioconjugate prepared (MoS_2_-ORFTDN-CNDsTy) is drop casted on CSPE electrodes and faced with the SARS-CoV-2 ORF1ab specific sequence. The hybridization event is detected following the CNDsTy electrochemical response by Differential Pulse Voltammetry (DPV).

### Bioconjugate preparation and electrochemical DNA biosensor development

The bioconjugate was synthesized by incubating the ORFTDN with the CNDsTy as described in detail in the experimental section. Briefly, equal volumes of ORFTDN and CNDsTy were mixed and incubated for 1 h at room temperature in the dark. Next, a washing process was followed to remove unbound molecules and finally obtain the mixture of CNDsTy-labelled ORFTDN, thanks to the interaction of the CNDsTy and the DNA. The next step is the immobilization of the ORFTDN-CNDsTy on the MoS_2_ due to the interaction between the thiol groups of the basal vertices of the ORFTDN and the sulphur vacancies of the MoS_2_ nanolayers, obtaining the MoS_2_-ORFTDN-CNDsTy bioconjugate. Finally, the mixture was centrifuged to remove the unbound molecules. Once the bioconjugate was prepared it was characterized by different techniques such as spectrophotometry, fluorescence, and cyclic voltammetry to assess its correct preparation.

Experimental conditions for bioconjugate preparation were optimized and are shown in Fig. 7 SI. It can be observed that using an excess of CNDsTy with a concentration of 100 µM of ORFTDN confirms the electrochemical label of the bioconjugate (Figure S7B of SI). Bioconjugate stability was also tested spectrophotometrically using different stocks prepared on different days (Fig. 7A of SI). As can be observed in the UV–Visible (Figure S7A), for a 1:4 dilution of different bioconjugate stocks in water, the same absorbance is obtained, so it can be confirmed that the concentration of the MoS2-ORFTDN-CNDsTy prepared following the protocol described is always the same. Finally, regarding the transducer selection, different electrodes such as CSPE and AuSPE were studied, see Figure S7B. Based on the electrochemical signals obtained CSPEs were chosen as transducers based on the best electrochemical signal obtained.

**Fig. 1:**
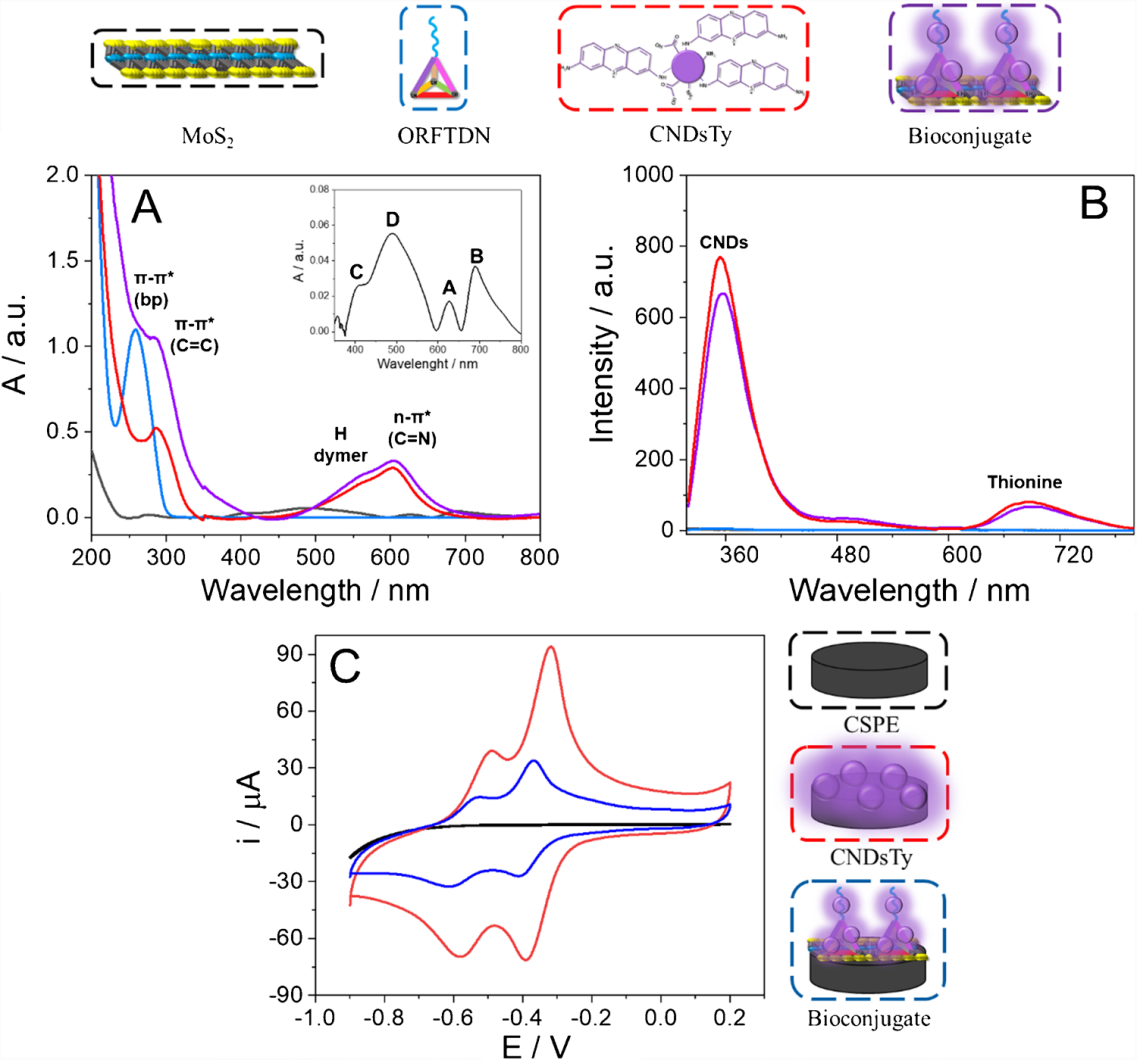
UV–Visible spectra (**A**) and fluorescence emission spectra (excited at 300 nm) (**B**) of: MoS_2_ in 2-propanol/water (7:3; v/v) (black line, inset), 500 nM ORFTDN in TM buffer solution (blue line), 88.4 µg/mL CNDsTy in water (red line), and MoS_2_-ORFTDN-CNDsTy 1:4 dilution in water (purple line). Cyclic voltammetry (**C**) of a bare CSPE (black curve), a CSPE modified with 2.83 mg/mL CNDsTy (red curve), and a CSPE modified with the bioconjugate, MoS_2_-ORFTDN-CNDsTy (blue curve), in PB 0.1 M pH 7.0 buffer. Scan rate: 10.0 mV s^−1^

Figure 1 shows the UV–Visible spectra (Fig. 1A) and fluorescence emission spectra (Fig. 1B) of the bioconjugate (MoS_2_-ORFTDN-CNDsTy) and the nanomaterials independently (MoS_2_, ORFTDN, and CNDsTy). Figure 1A portraits the typical absorption bands of the excitons A and B of the MoS_2_ (black line), at 627 and 690 nm, respectively. In addition, bands at 419 and 490 nm, corresponding to the excitons C and D of the MoS_2_ due to the higher energy transitions from the band edges of the colloid, were observed [25]. In the case of the ORFTDN spectrum (blue line), a maximum is observed around 258 nm, characteristic of the π–π* transition of DNA nitrogenous bases derived from pyrimidine and purine structures [26]. For the CNDsTy (red line), an absorbance peak at 285 nm is observed, due to the π–π* transition of the C = C bonds of the CNDs and the π–π* transitions of the aromatic ring of the phenothiazine [27, 28]. The covalent bonding between thionine and CNDs is confirmed by the band placed at 603 nm, close to the maximum of thionine at 599 nm. This peak corresponds to the n–π* transition of the C = N bond and to the monomeric thionine, while the shoulder at 559 nm appears due to the formation of H-type dimers of the dye [29, 30]. Spectrum of the bioconjugate MoS_2_-ORFTDN-CNDsTy (purple line) shows all the peaks mentioned above. Peaks at 258 and 285 nm from the ORFTDN and CNDsTy, respectively, overlap into a single band at 280 nm. In addition, the characteristic band of the thionine of the CNDsTy is observed at 608 nm.

From the fluorescence emission spectra (excited at 300 nm) of Fig. 1B, it can be observed that no emission is registered for both the MoS_2_ and the ORFTDN (black and blue line, respectively). On the other hand, two maximum emission peaks are observed for the CNDsTy and for the bioconjugate (red and purple lines, respectively). The emission band at 354 nm corresponds to the carbon nanodots, and the band at 687 nm is characteristic of the thionine molecule [28, 31]. These results point to the correct preparation of the MoS_2_-ORFTDN-CNDsTy bioconjugate.

We have also carried out an electrochemical characterization of the bioconjugate based on the redox properties of the CNDsTy. Figure 1C shows the cyclic voltammograms of a bare CSPE (black curve) and the MoS_2_-ORFTDN-CNDsTy bioconjugate on CSPE (blue curve). As control, the redox signal of the CNDsTy on CSPE was also registered (red curve). No redox process is observed for the bare CSPE, as no electroactive specie is immobilized on the surface of the electrode. After CSPE surface modification with CNDsTy (CSPE/CNDsTy), two reversible redox processes, characteristic of the thionine molecules embedded on the carbon nanodots nanostructure, are observed at a formal potential of − 0.32 V and − 0.49 V. One electron is exchanged in each of the redox processes. When the MoS_2_-ORFTDN-CNDsTy bioconjugate is immobilized on a CSPE (CSPE/MoS_2_-ORFTDN-CNDsTy), the characteristic redox behaviour of the CNDsTy is observed. These results evidenced again the labelling of the bioconjugate with the CNDsTy.

To confirm the correct immobilization, the platform was characterized by different techniques such as scanning electron microscopy (SEM) with energy dispersive X-ray spectroscopy (EDX), atomic force microscopy (AFM), fluorescence microscopy, and Raman spectroscopy.

**Fig. 2:**
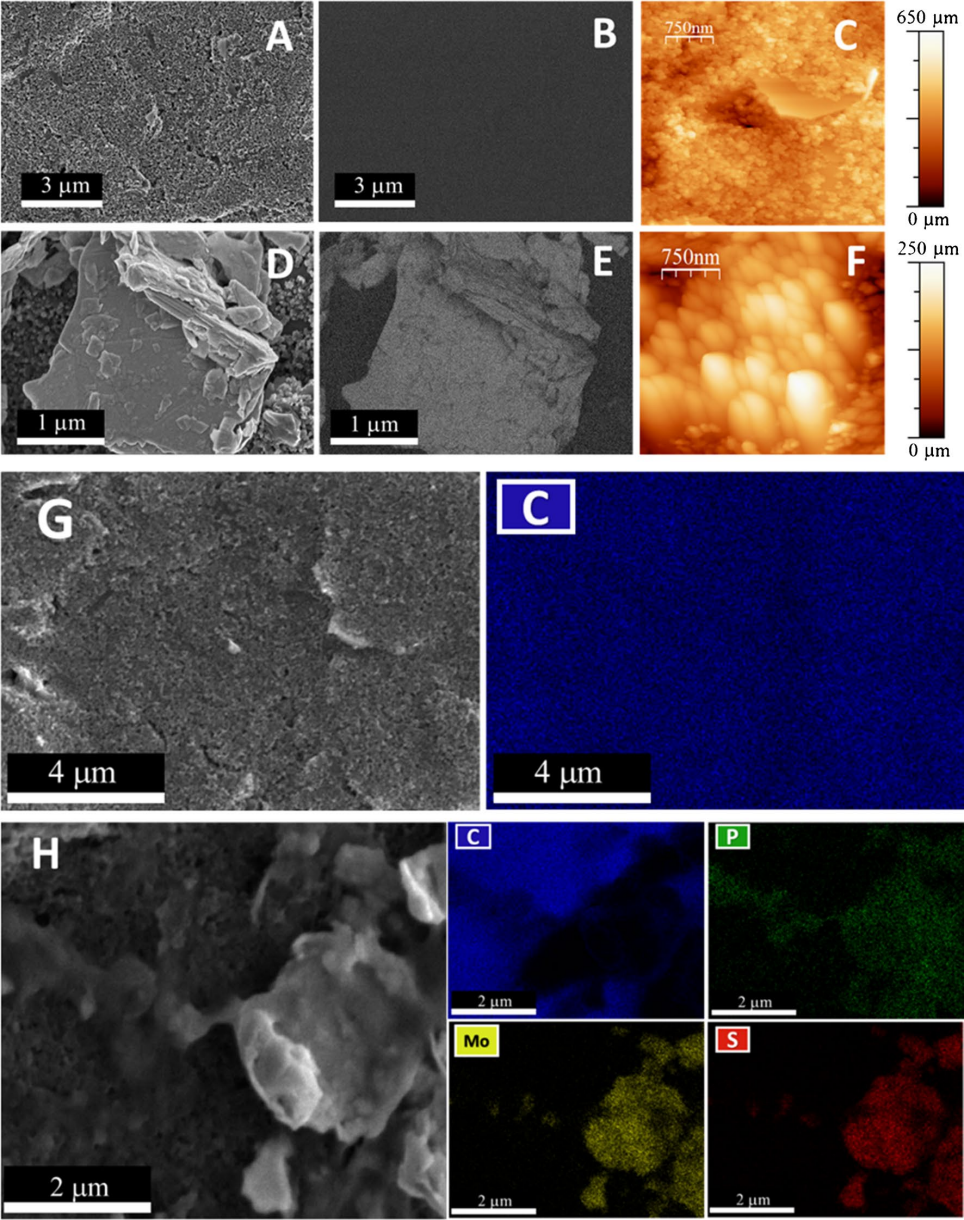
Secondary electrons images (**A, D**) and backscattered electrons images (**B, E**), AFM images (**C, F**), and EDX elemental mapping (**G, H**) of CSPE (**A–C, G**) and CSPE/MoS_2_-ORFTDN-CNDsTy (**D–F, H**)

Figure 2 presents a detailed comparison of SEM (Fig. 2A, B, D, E, G, H) and AFM (Fig. 2C, F) images of bare CSPE (Fig. 2A–C, G) and CSPE after the immobilization of the MoS₂-ORFTDN-CNDsTy bioconjugate (Fig. 2D–F, H).

The secondary electron images provide insights into the sample’s surface topography. For the bare CSPE (Fig. 2A, G), a homogeneous surface composed of carbon grains is observed. In contrast, after the immobilization of the bioconjugate (Fig. 2D, H), distinct changes in the electrode’s topography become evident, showing MoS₂ nanoflakes.

Backscattered electron images (Fig. 2B, E) further highlight the differences in the atomic composition before and after bioconjugate immobilization. The lighter regions in Fig. 2E, compared to the uniform carbon surface in Fig. 2B, confirm the presence of the MoS₂.

The AFM images (Fig. 2C, F) reveal topographical changes in the electrode surface. Before immobilization (Fig. 2C), the CSPE displays a smooth, homogeneous surface. However, after the bioconjugate attachment (Fig. 2F), the surface is covered by a large agglomerate, corresponding to the MoS_2_.

To validate the contribution of each bioconjugate component, controls were conducted using a CSPE modified solely with MoS₂ (Figures S8A–S8C) and another modified with the MoS₂-ORFTDN heterostructure without labeling (Figures S8D–S8F). These controls demonstrate that the topography and morphology of the electrode surface evolve distinctly at each modification stage.

Finally, elemental analysis through EDX mapping (Fig. 2G, H) confirms that bare CSPE (Fig. 2G) is composed entirely of carbon, while the CSPE/MoS₂-ORFTDN-CNDsTy platform (Fig. 2H) reveals the presence and distribution of carbon (from the electrode surface), molybdenum and sulfur (from the MoS₂ nanoflakes), and phosphorus (primarily from the DNA phosphate backbone of ORFTDN). Notably, the phosphorus localization overlaps predominantly with molybdenum and sulfur, indicating that the ORFTDN is preferentially immobilized on the MoS₂ nanoflakes.

**Fig. 3:**
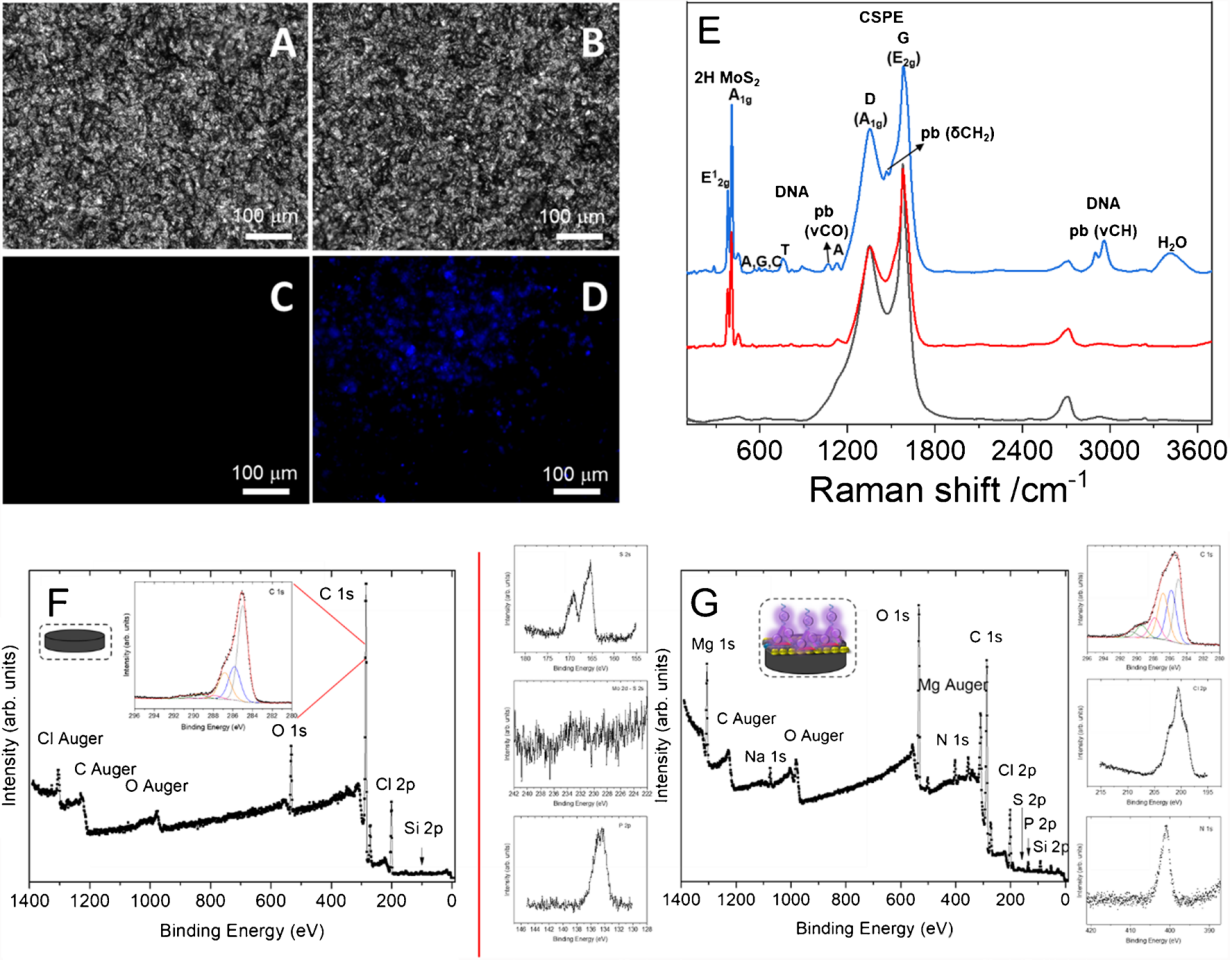
Bright field images (**A, B**) and fluorescence images (**C, D**) of a CSPE before (**A, C**) and after MoS_2_-ORFTDN-CNDsTy immobilization (**B, D**). Raman spectra (**E**) of a bare CSPE (black line), and of a CSPE modified with MoS_2_ (CSPE/MoS_2_, red line), or with the nanostructured bioconjugate (CSPE/MoS_2_-ORFTDN-CNDsTy, blue line). XPS survey spectra for the bare CSPE electrode (**F**) and for the nanostructured bioconjugate immobilized on CSPE (CSPE/MoS_2_-ORFTDN-CNDsTy) (**G**). In the insets, the most representative high-resolution core levels of each sample

Optical microscopy images were taken before (Fig. 3A, C) and after (Fig. 3B, D) MoS_2_-ORFTDN-CNDsTy immobilization, including both, bright field images (Fig. 3A, B) and fluorescence images (Fig. 3C, D). Fluorescence is only observed after the bioconjugate immobilization, due to the fluorescent properties of the CNDsTy. These results have been confirmed with the fluorescence images of the CSPE/MoS_2_ and CSPE/MoS_2_-ORFTDN controls, shown in Figures S9C and S9D of the Supporting Information respectively, where no fluorescent emission is observed. These experiments suggest the correct absorption of the MoS_2_-ORFTDN-CNDsTy bioconjugate on the electrode surface.

Raman spectroscopy was also used to confirm bioconjugate immobilization on the CSPE surface (Fig. 3E). As it is shown, the bare CSPE (black line) presents the characteristic D and G bands corresponding to the A_1g_ and E_2g_ active Raman modes of the carbon of the electrode, at 1351 cm^−1^ and 1581 cm^−1^, respectively [32, 33]. After bioconjugate immobilization (blue line), the characteristic guanine (594 and 672 cm^−1^), adenine (637, 672, and 1130 cm^−1^), cytosine (594 and 637 cm^−1^), thymine (672 and 759 cm^−1^), and phosphate backbone (759, 819, 890, 1062, 1468, 2900, and 2959 cm^−1^) signals of the ORFTDN appear [34]. These low intensity bands of the DNA embedded in the bioconjugate were confirmed by the Raman spectra of a CSPE modified with massive calf thymus DNA (dsDNA), as shown in Figure S10 of SI. Moreover, the in-plane E^1^_2g_ and the out-plane A_1g_ Raman modes of the 2H-polytype structure of the MoS_2_ (red line) appear at 379 and 404 cm^−1^.[35] All these results above described point to a correct immobilization of the nanostructured bioconjugate on the CSPE surface.

XPS measurements were also performed to confirm the bioconjugate immobilization on the CSPE. Figure 3F, G shows the survey spectra corresponding to the bare CSPE electrode and the CSPE/MoS_2_-ORFTDN-CNDsTy respectively. The insets show the most representative core levels of each sample. All the spectra are corrected with the C 1 s component of C–C/C–H bond, centred at 285.0 eV and taken as a binding energy reference. As can be observed in Fig. 3F, only the elements of the bare electrode are present; however, after bioconjugate immobilization on the CSPE (Fig. 3G), the elements of N, S, and P corresponding to the ORFTDN, MoS_2_, and CNDsTy, are observed. To confirm the peaks of the bioconjugate, Figures S11 and S12 of the SI show the XPS core levels obtained after MoS or ORFTDN immobilization on the CSPE. These results suggest the correct immobilization of the bioconjugate on the electrode surface.

### Electrochemical detection of SARS-CoV-2 ORF1ab DNA sequence

**Fig. 4:**
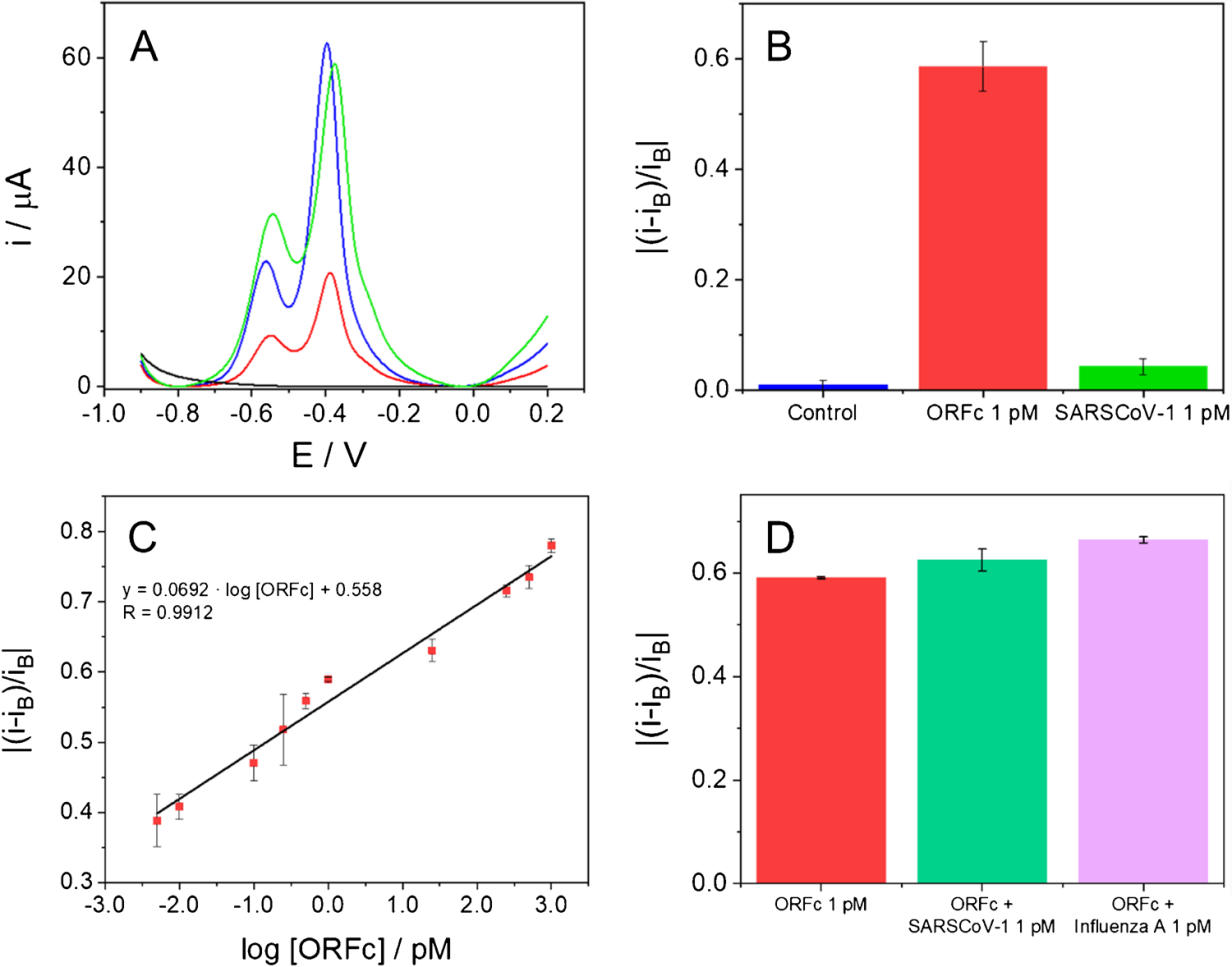
Differential pulse voltammograms (DPV) (**A**), and bar diagram of the normalized current intensity (**B**) obtained for a bare CSPE (blank line), and for the biosensing platform, before, CSPE/MoS_2_-ORFTDN-CNDsTy (blue line), and after the incubation with the ORF1ab specific sequence (ORFc, red line) or with a non-complementary sequence (SARS-CoV-1, green line) at 1.00 pM concentration, in PB 0.1 M pH 7.0. Oxidation peaks observed in blue, red, and green lines correspond to the current peak of CNDsTy. Scan rate: 10.0 mV s^−1^. Calibration plot (**C**) of the normalized current intensity registered versus the logarithm of the SARS-CoV-2 ORF1ab sequence (from 5.00 fM to 1.00 nM). Bar diagram of the normalized current intensity obtained for the ORF1ab sequence in the presence of other interfering virus sequences (SARS-CoV-1 and Influenza A) at 1.00 pM concentration (**D**). Data presented as mean ± standard deviation (*n* = 3)

After characterizing the biosensing platform, its ability to detect the virus from its genetic code was evaluated. In particular, as a model of virus, the ORF1ab SARS-CoV-2 specific sequence (ORFc), was studied. The incubation step with the ORFc sequence was optimized by following two different strategies, which consisted of pre-incubation of the analyte sequence with the bioconjugate and its subsequent immobilization on the CSPE surface, or pre-immobilization of the bioconjugate and subsequent incubation with the ORFc sequence. As only the second strategy showed differences in the electrochemical response of the platform in the absence or presence of the analyte, see Figure S7C, this strategy was chosen for the development of the biosensor. For that, the CSPE/MoS_2_-ORFTDN-CNDsTy platform was incubated with 10.0 µL of ORFc 1.00 pM for 1 h at 40 °C, in a humidity chamber under stirring. Then, differential pulse voltammograms of a bare carbon electrode (CSPE), and of the biosensing platform (CSPE/MoS_2_-ORFTDN-CNDsTy) before and after the incubation with the analyte (ORF1ab sequence), were recorded using PB 0.1 M pH 7.0 as electrolyte. As can be observed in Fig. 4A, no signal is registered for the bare CSPE (black curve) as no electroactive specie is immobilized on its surface. When the bioconjugate is deposited onto the CSPE (blue curve), the characteristic oxidation peaks of the CNDsTy at − 0.56 V and − 0.39 V were observed. A clear difference in the current intensity recorded at a potential of − 0.39 V before and after the hybridization event with the ORF1ab sequence is observed. Particularly, in the presence of the analyte, SARS-CoV-2 virus sequence (red curve), the current intensity seriously decreases. This phenomenon occurs due to the displacement of the redox indicator of the bioconjugate. CNDsTy is able to interact directly with the nitrogenous bases of the DNA, but when the hybridization event between the probe and the analyte takes place, the affinity of the probe with the complementary analyte sequence is higher than the electrostatic and intercalative interaction with the CNDsTy [36, 37].

With the aim of confirming that the differences observed on the current intensity registered become from the hybridization reaction between the probe and the specific ORF1ab sequence, the sensing platform (CSPE/MoS_2_-ORFTDN-CNDsTy) was incubated with a non-complementary sequence from SARS-CoV-1 virus. As shown in Fig. 4A, no signal difference is observed before (blue curve) and after the incubation with the SARS-CoV-1 sequence (green curve), since no hybridization with the probe has occurred. This result points to the selectivity of the biosensing platform to the SARS-CoV-2 virus.

Figure 4B shows the normalized current intensity obtained after the incubation with the ORF1ab sequence or with the SARS-CoV-1 one at the same concentration. The normalized signals were calculated from the difference between the signal recorded before (Blank, CSPE/MoS_2_-ORFTDN-CNDsTy) and after the hybridization reaction divided by the blank signal in absolute value (| (Hybridized signal − Blank signal) / Blank signal |). As it is expected when the biosensing platform is incubated with the complementary sequence (ORF1ab, red bar) the normalized signal obtained is higher than the one registered with the non-complementary sequence (SARS-CoV-1, green bar), as there was no hybridization reaction.

These results confirm the ability of the biosensing platform developed to detect a virus by its genetic code and its selectivity to the ORF1ab SARS-CoV-2 specific sequence.

After studying and demonstrating the ability of the biosensing platform to detect the SARS-CoV-2 ORF1ab specific sequence, we study the behaviour of the biosensing platform after the incubation with different ORF1ab sequence concentrations (from 5.00 fM to 1.00 nM). Figure 4C shows the normalized current intensity (calculated as in the selectivity study) versus the logarithm of the ORF1ab concentration. As can be observed, the normalized signal at − 0.39 V increases gradually on increasing concentrations of ORF1ab sequence and fits to the linear equation: Normalized signal = 0.0692·log [ORFc] + 0.558 (*R* = 0.9912). Values are the mean of three different measurements with a sensitivity of 0.069 a.u. log(fg^−1^ mL) and a percentage coefficient variation (CV) of 1.90%. The detection limit was found to be 5.00 fM.

The selectivity of the biosensor was also evaluated in the presence of some interfering DNA sequences specific to other viruses such as SARS-CoV-1 or Influenza A. Figure 4D shows the bar diagrams obtained for the normalized current intensity signals of the biosensor incubated with the ORF1ab sequence at 1.00 pM concentration (red bar), or with the mixture of ORF1ab and the interfering virus DNA sequences at the same concentration (SARS-CoV-1, green bar and Influenza A, purple bar). There are no differences on the signal registered, so the biosensor response is not affected, and no cross-interference is observed.

The response of the developed biosensor stored at 4 °C for a 1.00 pM concentration of the SARS-CoV-2 ORF1ab sequence remains practically constant for 50 days, as shown in Figure S13 of the SI.

#### SARS-CoV-2 ORF1ab DNA sequence detection in infected patients

**Fig. 5:**
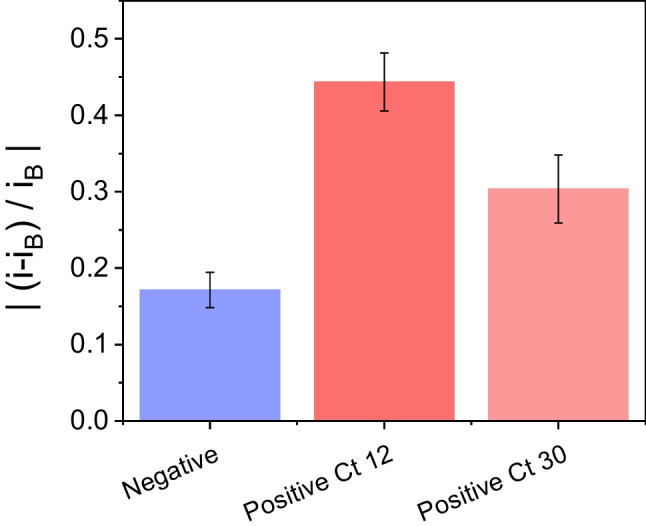
Bar diagram of the normalized current intensity obtained after incubating the CSPE/MoS_2_-ORFTDN-CNDsTy platform with 10.0 µL of nasopharyngeal sample solutions from three patients: Negative sample (blue bar), high viral load sample, Ct 12 (dark red bar), and low viral load sample, Ct 30 (light red bar)

The interest in the search of new alternative detection techniques to those commonly used for the detection of all types of pathogens has grown significantly in recent years with the emergence of new viruses and bacteria causing diseases with high levels of spread. Thus, given the success results obtained for the DNA biosensor developed in this work for the specific detection of the SARS-CoV-2 ORF1ab sequence, we went a step forward and we study the capacity of the sensing platform to detect the virus in nasopharyngeal human samples testing its applicability as an alternative method to the classical detection ones. Nasopharyngeal samples from a non-infected patient and two SARS-CoV-2 infected patients with different viral load have been provided by the “Instituto Ramón y Cajal de Investigación Sanitaria” (IRYCIS) of the Autonomous Community of Madrid. Therefore, the sensing platform (CSPE/MoS_2_-ORFTDN-CNDsTy) was incubated with 10.0 µL of infected samples with high viral load (Ct 12) and with low viral load (Ct 30), and a non-infected sample as control. The normalized current intensity signal registered for each patient is presented on the bar diagram of Fig. 5. As can be observed, the negative patient presents the smallest normalized signal value, since not being infected by the virus, the hybridization reaction does not occur. Furthermore, comparing the normalized signals obtained for the infected patients, with high viral load (Ct 12, dark red) and with low viral load (Ct 30, light red), a clear difference in the signal is observed, since the concentration of the virus present in the sample is different. Being higher the normalized current intensity registered for the high viral load sample, as expected with the calibration plot. These results allow us to affirm that the biosensor developed is capable of detecting the SARS-CoV-2 virus in infected patient samples, and it is also capable to differentiate between patients with different viral loads, which demonstrates its potential application as an alternative virus detection method to those currently used.

Table 1 shows other electrochemical biosensors described in the literature to detect the SARS-CoV-2 ORF1ab sequence. The biosensor developed in this work presents competitive analytical parameters comparing with those reported in the literature for similar platforms [23, 38, 39]. This proves that the preparation and use of bioconjugates, in this case the MoS_2_-ORFTDN-CNDsTy bioconjugate, for the development of electrochemical biosensors is a great, fast, and simple practical alternative for virus detection.

**Table 1:** Comparative table for other electrochemical DNA biosensors for SARS-CoV-2 virus detection by its genetic code

Biosensor	Based on	Method	LOD	Reference
Electrochemical DNA biosensor (ORF1ab sequence)	MoS_2_-ORFTDN-CNDsTy bioconjugate	DPV	5.00 fM	This work
Electrochemical DNA biosensor (ORF1ab sequence)	MoS_2_/CSPE	DPV	1.01 pM	Martínez-Periñán et al. [23]
Paper-based electrochemical DNA sensor (SARS-CoV-2 N gene)	Pyrrolidinyl peptide nucleic acid-based electrochemical paper-based analytical device (PNA-based ePAD)	Amperometry	1.00 pM	Lomae et al. [38]
Electrochemical DNA biosensor (RdRp sequence)	AA/AuNTS/CSPE	DPV	22.2 fM	Del Caño et al. [39]

To demonstrate the great applicability of the developed biosensor and the versatility offered by the use of the bioconjugate for the detection of different analytes of great clinical interest from their genetic code, a biosensor was prepared for the specific detection of the BRCA1 gene associated with breast cancer. For this purpose, a new TDN was synthesized in which the probe of the upper vertex was modified by a specific and complementary sequence to the BRCA1 gene. The bioconjugate was then prepared following the same protocol proposed in this work and immobilized on a CSPE (CSPE/MoS_2_-BRCA1TDN-CNDsTy). Both incubation and detection of the hybridization event were carried out under the same experimental conditions described in the experimental section. Figure S14 of the SI shows a bar graph of the maximum current intensity recorded at the − 0.4 V potential characteristic of CNDsTy before (blue bar) and after (red line) hybridization of the biosensing platform with the BRCA1 gene. As can be observed, there are differences between the signal obtained before and after hybridization, so it can be stated that the biosensor developed from a bioconjugate is capable of detecting the specific sequence of the BRCA1 gene and that it is applicable to the detection of any analyte of interest, provided that the TDN synthesized has in its structure the specific probe for that analyte.

## Conclusions

A new biconjugate based on MoS_2_ nanoflakes, tetrahedral DNA nanostructures carrying the specific SARS-CoV-2 ORF1ab probe sequence, and thionine-carbon nanodots as redox indicators of the hybridization event has been prepared and deeply characterized. The prepared bioconjugate has been applied for the development of a new fast, simple, selective, and sensitive electrochemical DNA biosensor for virus detection. In particular, SARS-CoV-2 virus was selected as analyte due to the great interest in the last few years to developed alternative detection methods easier, faster, more effective, and more sensitive than the classical ones. The use of nanomaterials and nanostructures such as the MoS_2_ and the ORFTDN in the bioconjugate preparation and biosensor development allows to achieve a limit of detection of 5.00 fM with a sensitivity of 0.0692 a.u. · log(fg^−1^ mL), a stability of 50 days, and a higher selectivity in the presence of other interfering viruses. Furthermore, the biosensor developed is able to detect the specific SARS-CoV-2 ORF1ab sequence in nasopharyngeal human samples of infected patients with high (Ct 12) and low (Ct 30) viral load. Hence, the prepared bioconjugate and the developed biosensor can be proposed as an alternative methodology for virus detection.

## Supplementary Information

Below is the link to the electronic supplementary material.

ESM1 (DOCX 10.2 MB)

## Data Availability

No datasets were generated or analysed during the current study.
