# Supplementary material for: MoS₂-DNA tetrahedral bioconjugate for high-performance DNA biosensors: application in viral infection diagnostics
Source: Mikrochim Acta. 2025 Mar 11;192(4):221. doi: 10.1007/s00604-025-07084-2 (PMC11897093; doi:10.1007/s00604-025-07084-2)
Supplement: Supplementary file 1 — ESM1 (DOCX 10.2 MB) [file 604_2025_7084_MOESM1_ESM.docx]

**Supporting Information**

**MoS₂-DNA Tetrahedral Bioconjugate for High-Performance DNA Biosensors: Application in Viral Infection Diagnostics.**

*Estefanía Enebral-Romero^a,b^, Emiliano Martínez-Periñán^b,c^, David López-Diego^d^, Mónica Luna^d^, Marina Garrido^a^, Cristina Navío^a^, Emilio M. Pérez^a^, Encarnación Lorenzo^a,b,c^ and Tania García-Mendiola^b,c*^*

^a^ IMDEA-Nanociencia, Ciudad Universitaria de Cantoblanco. 28049, Madrid (Spain).

^b^ Departamento de Química Analítica y Análisis Instrumental. Universidad Autónoma de Madrid. 28049, Madrid (Spain).

^c^ Institute for Advanced Research in Chemical Sciences (IAdChem). Universidad Autónoma de Madrid. 28049, Madrid (Spain).

^d^ Instituto de Micro y Nanotecnología IMN-CNM, CSIC (CEI UAM+CSIC), Isaac Newton 8. Tres Cantos. 28760, Madrid (Spain).

*Corresponding author: [tania.garcia@uam.es](mailto:tania.garcia@uam.es)

**Experimental section**

***Reagents and materials***

Sodium chloride, monobasic sodium phosphate, dibasic sodium phosphate, magnesium chloride, tris (hydroxymethyl)aminomethane (TRIS, NH_2_C(CH_2_OH)_3_), L-arginine, 3,3’-diamino-N-methyldipropylamine, thionine acetate salt, 1,4-Dithiothreitol (DTT), tetrahedral DNA sequences and interferent DNA sequences (SARS-CoV-1 and Influenza A), shown in Table 1, and bulk MoS_2_ powder (<2 µm, 98%) were purchased by Merck (<https://www.merckgroup.com/>).

Nasopharyngeal human samples from a non-infected patient and from two SARS-CoV-2 infected patients with low and high viral load were purchased by the Instituto Ramón y Cajal de Investigación Sanitaria (IRYCIS), in accordance with the “Ley de Investigación Biomédica 15/2007” and “Real Decreto de Biobancos 1716/2011”. All the patients consent and approved the sample study by “Comité de Ética de la Investigación con Medicamentos del Hospital Universitario Ramón y Cajal” (REF: 127-21).

**Table 1.** Oligonucleotide sequences used in this work

| **Oligonucleotide sequences** | | **Named** |
| --- | --- | --- |
| Tetra A- ORF 1ab SARS-CoV-2 sequence | 5’-CCA TAA CCT TTC CA TTTTTTTTTT ACA TTC CTA AGT CTG AAA CAT TAC AGC TTG CTA CAC GAG AAG AGC CGC CAT AGT-3’ | ORF-Tetra A |
| Tetra B | 5’-HS-(CH_2_)_6_-TAT CAC CAG GCA GTT GAC AGT GTA GCA AGCTGT AAT AGA TGC GAG GGT CCA ATA C-3’ | Tetra-B |
| Tetra C | 5’-HS-(CH_2_)_6_-TCA ACT GCC TGG TGA TAA AAC GAC ACT ACG TGG GAA TCT ACT ATG GCG GCT CTT C-3’ | Tetra-C |
| Tetra D | 5’-HS-(CH_2_)_6_-TTC AGA CTT AGG AAT GTG CTT CCC ACG TAG TGT CGT TTG TAT TGG ACC CTC GCA T-3’ | Tetra-D |
| Tetra A- BRCA1 gen sequence | 5’-GAC TCA CCT GCA ATA AGT TG TTTTTTTTTT ACA TTC CTA AGT CTG AAA CAT TAC AGC TTG CTA CAC GAG AAG AGC CGC CAT AGT-3’ | BRCA1-Tetra A |
| ORF 1ab complementary sequence | Analyte | ORFc |
| SARS-CoV-1 sequence | Interferent 1 | SARS-CoV-1 |
| Influenza A sequence | Interferent 2 | Influenza A |
| BRCA1 complementary sequence |  | BRCA1 |

***Experimental instruments***

The synthesis of the tetrahedral DNA nanostructure containing the ORF1ab SARS-CoV-2 specific sequence (ORFTDN) was performed in a MiniAmpPlus^TM^ Thermal Cycler.

ORFTDN fluorescence microscopy measurements were performed with an incident light microscope Axioskop 2 MAT (ZEISS) optical microscope, using a HBO 50W/AC L1 (OSRAM) mercury short arc lamp.

The exfoliation of bulk MoS_2_ was accomplished using a Vibracell 75115 (VC 505/VC 750) Bioblock Scientific ultrasonic probe. The centrifugation was performed in an Allegra X-15R Beckman Coulter centrifuge (FX6100 rotor).

For the synthesis of the CNDsTy a CEM Discover microwave system (Matthews (NC), USA) was used.

Transmission electron microscopy (TEM) images of the CNDsTy were registered with a JEOL JEM 2100 electron microscope. CNDsTy were deposited onto a Lacey carbon support film copper grid (400 mesh, Electron Microscopy Sciences).

Fourier transform infrared (FTIR) studies for the CNDsTy were performed with a Brucker IFS60v spectrometer. Spectra were recorded in a wavelength range from 5000 to 500 cm^-1^, using KBr pressed pellets of the CNDsTy synthetized and each of the components.

UV-Visible spectra were acquired with a UV-1900 from SHIMADZU spectrophotometer using a quartz cell.

Fluorometric experiments were done using a Cary Eclipse Varian spectrofluorometer and 1 cm optical pitch quartz cuvettes.

A Bruker Senterra confocal Raman microscope (Bruker Optic, Ettlingen, Germany, resolution 9-15 cm^-1^) with a NA 0.80 100X Olympus microscope objective, a laser excitation of 532 nm, and a power of 2 mW, was used for the Raman spectroscopy characterization of the MoS_2_ synthesized and the electrochemical DNA biosensor developed. Raman spectra are the average of the measures performed in different regions of the sample.

Atomic Force Microscopy (AFM) images were obtained with a Nanotec Electronic AFM system, using the jumping mode and silicon cantilevers (PPP-FM Nanosensors, 2.8 N/m nominal spring constant and 75 kHz resonant frequency). For the data acquisition and the image processing the WSxM software was used.[13]

SEM-EDX experiments were performed with a Scanning Electron Microscope VERIOS 460 from FEI, at 2 kV and with a current of 13 pA.

Fluorescence microscopy was carried out with an incident light microscope Axioskop 2 MAT (ZEISS), using a mercury short arc lamp HBO 50 W/AC L1 (OSRAM).

Electrochemical measurements were executed in an Autolab/PGSTAT 10 potentiostat from EcoChemie using the GPES 4.9 software. Differential Pulse Voltammograms baseline corrections were performed with the GPES 4.9 program. The Carbon Screen-Printed electrodes (CSPEs) used as transductors and the Screen-Printed electrode connector used as interface in this work were provided by Metrohm ([www.metrohm.com/es-es](http://www.metrohm.com/es-es)). A home-made electrochemical cell was used for the CNDsTy cyclic voltammetry characterization.

X-Ray photoelectron spectroscopy (XPS) measurements for the core level analysis were performed using as exciting photoelectron source a monochromatic Al Kα line (hν = 1486.7 eV). All the experiments were done under Ultra High Vacuum conditions, with a base pressure of 5×10^-10^ mbar. SPHERA-U7 hemispherical energy analyzer (20 eV pass energy, and resolution of 0.6 eV) was used to collect the emitted photoelectrons. A Flood Gun, (FG-500, Specs, 3 eV energy electrons and 40 μA) was used to compensate the built-up charge on the sample surface and the C 1s centered at 285.0 eV was taken as a binding energy correction.

All the solutions were prepared using the Purified water Millipore Milli-Q system (18.2 MΩ·cm). Nüve OT012 autoclave was used to sterilize all the solutions and materials.

***Methods***

***ORFTDN Cryo-EM characterization***

A two-steps process was followed for the Cry-EM ORFTDN characterization. First the ORFTDN Cryo-EM grids were prepared and vitrified with a Vitrobot Mark IV (FEI). Then Quantifoil <cu/Rh 1.2/1.3 300 mesh grids were glow-discharged at 15 mA for 1 minute and aliquots of 3 μL of the sample were added to the grids, dried for 3 seconds at 95% humidity and 20 °C, and immersed into liquid ethane. Finally, after sample screening, data was collected with a 200 kV FEI Talos Arctica equipped with a Falcon II direct electron detector cryo-EM at the Centro Nacional de Biotecnología (CNB-CSIC). 844 movies of ORFTDN were recorded with a defocus range from −1.7 to −3.3 μm and at a nominal magnification of 120,000X (0.855 Å/pixel, pixel size). All the movies were then divided into 60 frames with a total exposure of 31 second and a total dose of 30.9 e^−^/Å^2^ for each movie. The second step of the process consisted of cryo-EM data processing. For that, the collected movies were dose weighted and motion corrected with a Motion Cor2.[1] Additionally, cryoSPARC program was used for data processing.[2] gCTF estimation was then done on the dose weighted and imported aligned micrographs.[3] Particle selection was performed with a crYOLO.[4] Finally, the extracted particle stacks were cleaned by several interactive 2D classification and 2D class selection rounds.

***ORFTDN gel electrophoresis characterization***

The synthesized ORFTDN was characterized by a non-denaturing polyacrylamide gel electrophoresis (PAGE, 12.5%) using TBE as buffer at a constant current of 5 mA for 15 minutes and 10 mA for 2.5 hours at room temperature.

**Results and discussion**

***MoS_2_ characterization***

MoS_2_ flakes were characterized by Raman and UV-Visible spectroscopy (see Figure S1). Raman spectrum of the powder MoS_2_ of Figure S1A show the characteristic E^1^_2g_ and A_1g_ active Raman modes of the nanomaterial at a Raman shift of 385 and 405 cm^-1^, respectively.[5] UV-Visible spectrum shown in Figure S1B displayed the typical A and B excitons bands of the MoS_2_ at a wavelength of 624 and 685 nm, respectively. Furthermore, C and D excitons characteristic of the band edges high energy transitions, are observed at 488 and 411 nm, respectively.[6] These results confirm the 2H-polytype MoS_2_.


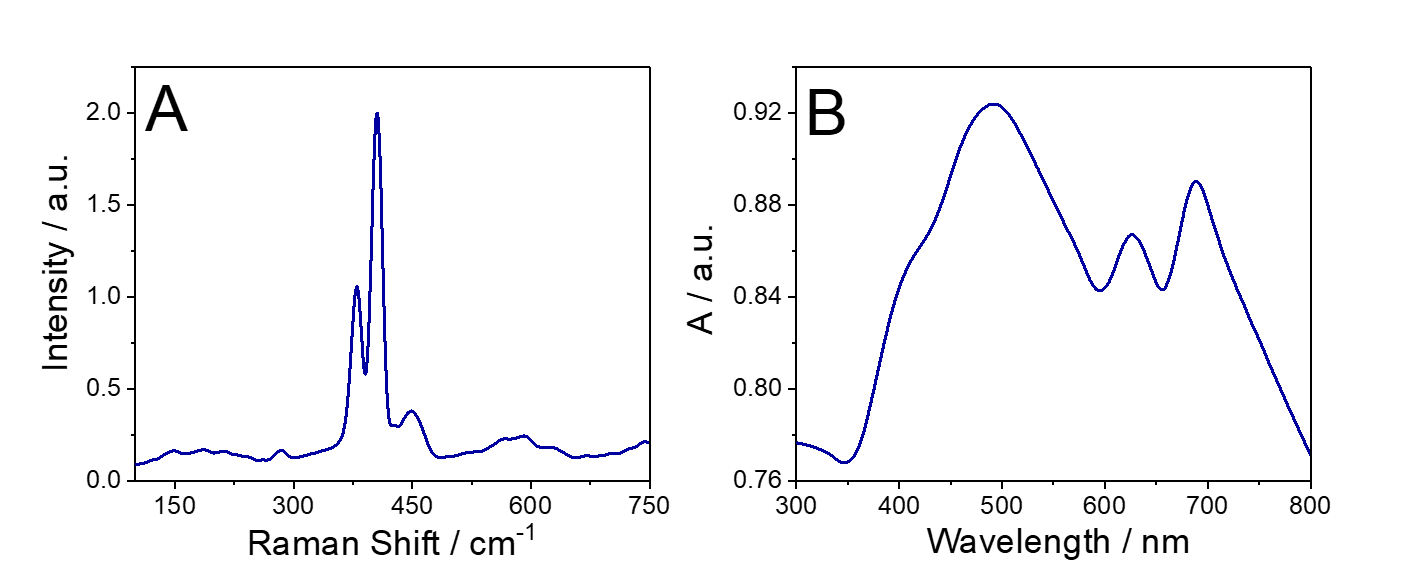


**Figure S1.** Raman spectrum (**A**) obtained for an exfoliated powder MoS_2_ (laser excitation of 532 nm), and UV-Visible spectrum (**B**) of exfoliated MoS_2_ in 2-propanol/water (7:3; v/v).

***Thionine modified carbon nanodots (CNDsTy) characterization***

Once the CNDsTy were synthesized, they were characterized by different microscopic, spectroscopic and electrochemical technics such as transmission electron microscopy (TEM), Fourier transform infrared (FTIR), UV-Visible spectroscopy, cyclic voltammetry (CV), and X-Ray photoelectron spectroscropy (XPS).

First, to estimate the CNDsTy size distribution, measurements of the average size of 120 CNDsTy were performed. As can be observed in the histogram of Figure S2, the mean size of the CNDsTy synthesized is around 3.1 nm, ranging from 1.5 to 5.0 in diameter.

**Figure S2.** Diameter histogram obtained from TEM measurements of 120 CNDsTy.

After estimating the CNDsTy mean size of 3.1 nm, it was assessed by TEM image (Figure S3A), that they are quasi-spherical nanoparticles.

Figure S3B shows the FT-IR spectrum of the prepared CNDsTy, and the independent elements, carbon nanodots (CNDs) and the acetate thionine (Ty). CNDs spectrum (black line), display the characteristic band corresponding to the C-O stretching vibration at 1071 cm^-1^. Bands corresponding to the C-N bond, and the C=N stretching vibration appears at 1384 and 1457 cm^-1^, and 1562 cm^-1^, respectively. C=O stretching vibration band is observed at 1644 cm^-1^. Band derived from the C-H bond stretching vibration appears at 2930 cm^-1^. Broad band centered at 3436 cm^-1^, is characteristic of the stretching band of OH and NH_2_. Thionine acetate spectrum (red line) shows the typical N-H bonding band, and the acetate anions of the thionine salt signals at 1617 and 2972 cm^-1^. Bands at 3147 and 3336 cm^-1^ correspond to the N-H primary amine symmetric stretching and the N-H asymmetric stretching, respectively. CNDsTy spectrum (blue line) indicates that there have been small conformational changes in the structure of the carbon nanodots after functionalization with thionine. The characteristic bands of the CNDs are observed, and in addition, the broad band at 3436 cm^-1^, typical of the amino groups of the thionine, and an increase in the 1617 cm^-1^ band associated with thionine, are observed.

UV-Visible spectroscopic studies (Figure S3C) of the CNDsTy, CNDs and Ty were performed. As can be observed in the spectra, no absorption band appears for the CNDs (black line). In the case of the Ty (red line), two absorption bands are observed at 285 nm and 598 nm, characteristic of the π – π* transition of the aromatic ring and the n–π* transitions of the C=N bond, respectively.[7] CNDsTy spectrum (blue line) shows a great light scattering due to the nanomaterial suspension. Absorption peaks at 285 nm attributed to the π-π* transition of the conjugated C=C bonds of the CNDs and the π – π* transition of the thionine aromatic ring,[8] and at 603 nm associated to the thionine covalently linked with the CNDs, are observed. As the UV-Visible spectra suggest, CNDs have been functionalized with the thionine molecule, obtaining a new nanomaterial with relatively different optical properties.

Electrochemical experiments were performed using PB 0.1M pH 7.0 as electrolyte solution. Figure S3D shows the cyclic voltammograms (CVs) recorded for a carbon screen-printed electrode (CSPE) modified with the CNDs, Ty or the CNDsTy. No redox process is observed for the CNDs (black line), as no faradaic process occur. For the thionine solution (red line), a reversible redox process is observed at a formal potential of -0.38 V, which involves two electrons. When the carbon nanodots are functionalized with the thionine, CNDsTy (blue line), two reversible redox processes are registered at a formal potential of -0.38 V and -0.54 V, with an exchange of one electron each. These results point again to the correct thionine functionalization of the CNDs, confirming that the thionine molecules are embedded in the carbon nanodots nanostructure.

XPS spectra of the N 1s and S 2p core levels of the CNDs (black line), Ty (red line) and CNDsTy (blue line) are presented on Figure S3E and S3F, respectively. As can be observed, for the CNDsTy, due to the presence of the sulphur atom, a shift on the N 1s peak of the CNDs towards higher binding energies is observed, which points to the correct functionalization of these with thionine.

**Figure S3.** TEM image (**A**) of the CNDsTy. FT-IR (**B**), UV-Visible spectra (**C**) and Cyclic Voltammetry (**D**) in PB 0.1M pH 7.0 at a scan rate of 100mV·s^-1^ of 0.283 mg·mL^-1^ CNDs (black line), 50 µM Thionine acetate (red line) and 0.283 mg·mL^-1^ of CNDsTy (blue line). XPS core levels spectra of N 1s (**E**) and S 2p (**F**) of CNDs (black line), Thionine acetate (red line) and CNDsTy (blue line).

***Study of the interaction of CNDsTy with DNA***

Spectrophotometric and fluorimetric titrations were performed to study the interaction of the prepared CNDsTy with DNA, in which the concentration of the CNDsTy (0.280 mg·mL^-1^) was fixed and the concentration of each single and double stranded calf thymus DNA (ssDNA and dsDNA) was increased from 10.0 µM to 200 µM in both water and 0.1M phosphate buffer pH 7.0 (PB). Figure S4 shows the UV-Visible spectra recorded for dsDNA (Figures S4A and S4C) and ssDNA (Figures S4B and S4D) in both water (Figures S4A and S4B) and PB (Figures S4C and S4D). As it can be observed, as the concentration of DNA increases, the absorbance progressively decreases at the absorption maximum of the CNDsTy at 600 nm, known as hypochromic effect. A shift to longer wavelengths, bathochromic effect, is also observed. These variations are more accused in the case of dsDNA. With the absorbance values recorded, the binding constants (K_b_) were calculated from the Becker and Meehan equation, and values of 7.05·10^5^ M^-1^ and 1.40·10^4^ M^-1^ for ds and ssDNA, respectively, were obtained. From the effects observed in the UV-Visible spectra and the K_b_ values it can be verified that there is a change in the local polarity around the chromophore that is given by the strong interaction that exists between the CNDsTy and the pyrimidine and purine bases of the DNA, and that this strong interaction is given mainly by the intercalation of the CNDsTy between the base pairs of the DNA, thanks to the aromatic ring structure presented by the thionine molecule of the CDNsTy. Furthermore, the difference in K_b_ values between dsDNA and ssDNA suggests that the CNDsTy have a higher affinity for dsDNA. To study how ionic strength could affect the interaction, titrations were performed in PB (Figures S4C and S4D), and the binding constants were calculated, obtaining values of 2.00·10^5^ M^-1^ and 1.00·10^4^ M^-1^ for dsDNA and ssDNA, respectively. The values are very similar to those obtained in water, which suggests that the increase in ionic strength does not affect the interaction, confirming that the interaction is mainly by intercalation and not by electrostatic interaction.


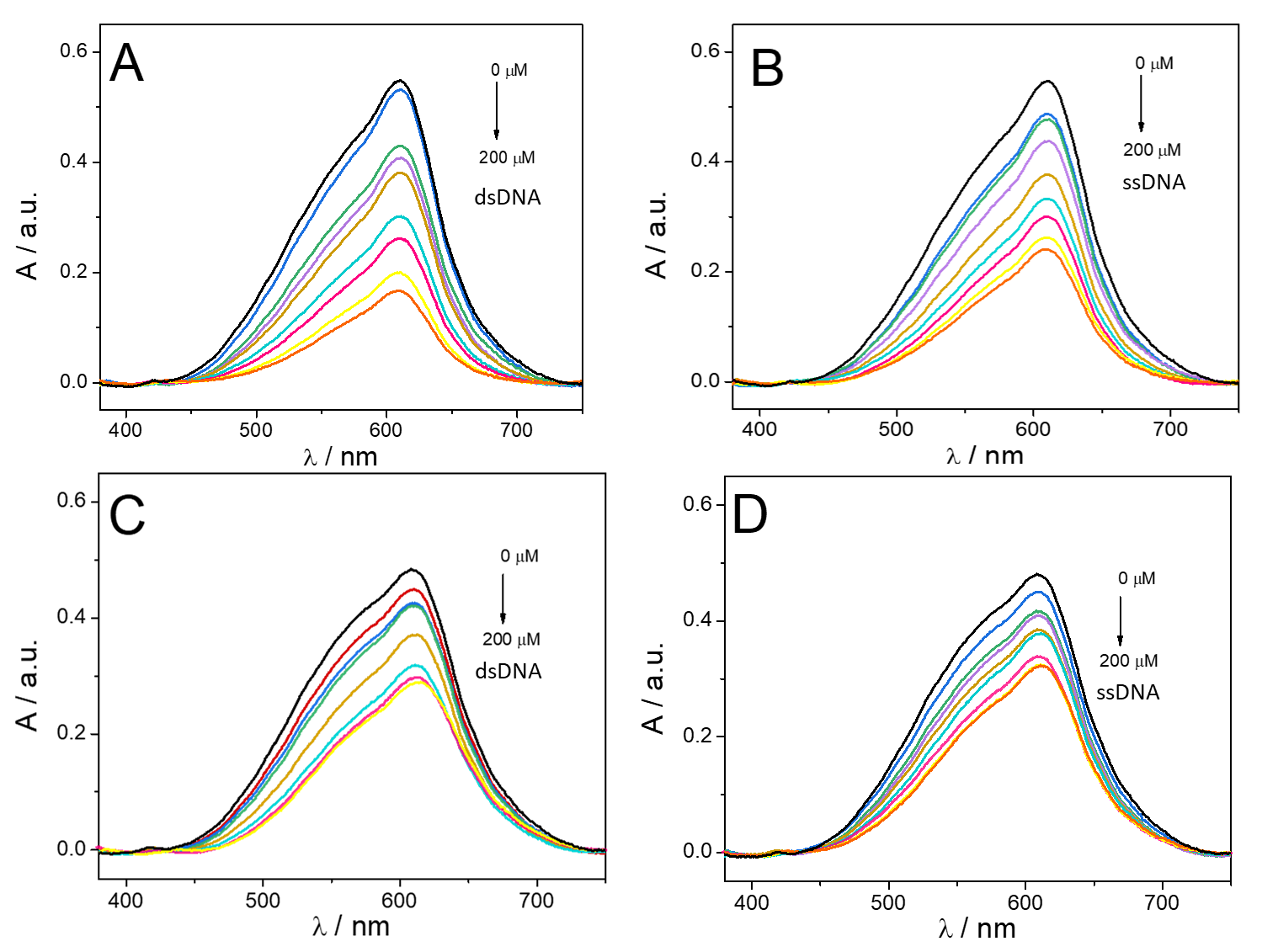


**Figure S4.** UV-Visible spectra of 0.280 mg·mL^-1^ CNDsTy in absence (black line) and in presence of increasing concentrations of dsDNA (**A** and **C**) or ssDNA (**B** and **D**) in water (**A** and **B**) and in 0.1 M phosphate buffer pH 7.0 (PB) (**C** and **D**).

For fluorescence titrations, emission spectra were recorded in water after excitation at 300 nm, as shown in Figure S5. By studying the emission maximum of the CNDsTy at 355 nm it can be observed how as the DNA concentration increases, the intensity in fluorescence emission increases progressively. The binding strength (K_sv_) for both dsDNA and ssDNA has been calculated from the Stern-Volmer equation, and values of 1.05·10^4^ and 5.20·10^3^ M^-1^ were obtained for each of them respectively. From the observed effect on the fluorescence emission and the calculated K_sv_ it can be stated that CNDsTy interact with DNA by intercalation and have a higher affinity for dsDNA, as in the absorbance titrations.


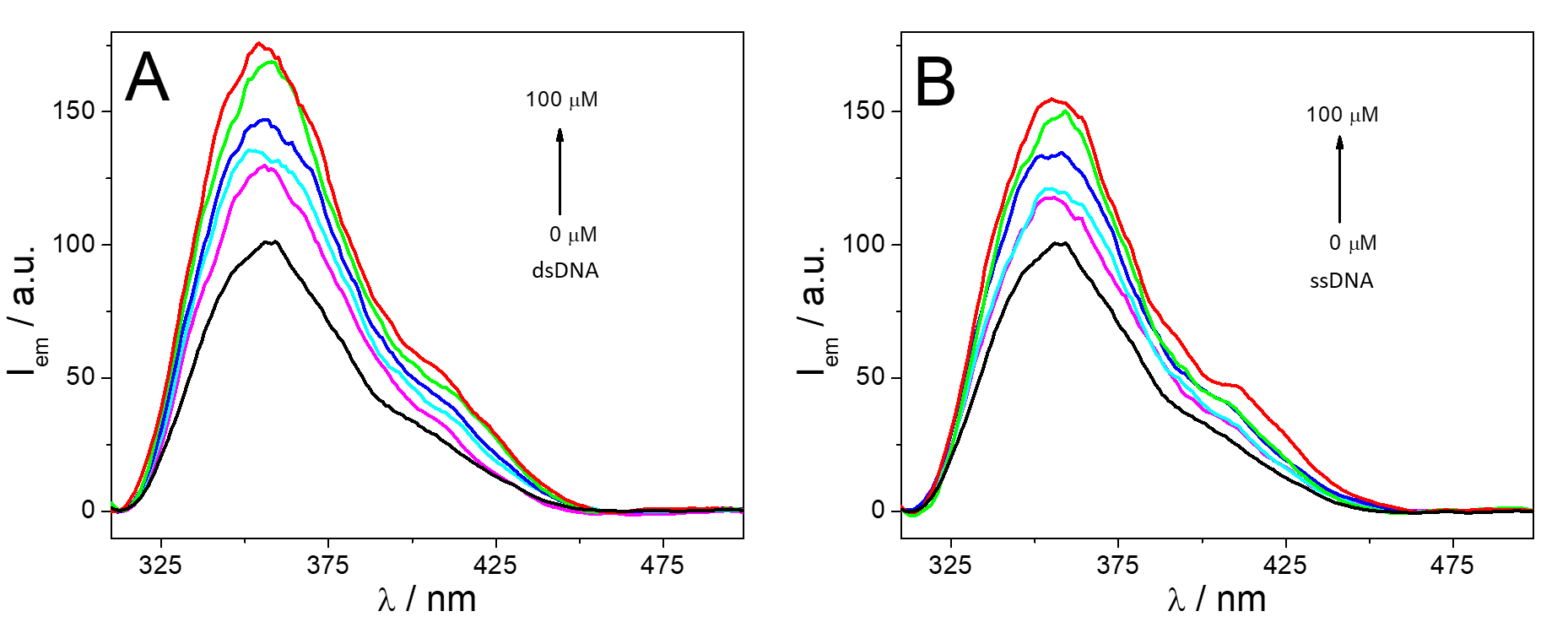


**Figure S5.** Fluorescence emission spectra (excited at 300 nm) of CNDsTy in absence (black line) and in presence of increasing concentrations of dsDNA (**A**) and ssDNA (**B**) in water.

***Tetrahedral DNA nanostructure synthesis and characterization***

The tetrahedral DNA nanostructure synthesized in this work was prepared from three 50-base thiolated DNA sequences and a 78-base DNA sequence containing the specific ORF1ab SARS-CoV-2 one (ORFTDN). The process followed for the synthesis is based on the complementarity of the nitrogenous bases, thus forming the tetrahedral nanostructure with the three thiol groups on the basal vertices and the analyte sequence on the top vertex, as shown in Figure S6A. The complementary fragments of each sequence are composed by 17 nitrogenous bases and are highlighted in different colours in Table 1 and Figure S6A.

The ORFTDN synthesis is divided in various steps with a controlled temperature drop, depending on the melting temperature (T_m_) of each complementary fragments, thus avoiding undesirable hybridizations. The formula used for the calculation of the T_m_ temperatures is T_m_ =64.9+41·(G+C-16.4)/L, being C and G the number of cytosine and guanine bases, and L the total number of bases (17 bases).

Once the ORFTDN were synthesized, they were deeply characterized to confirm the tetrahedral conformation of the nanostructure. They were characterized by optical microscopic techniques such as bright field and florescence, Cryogenic Electron Microscopy (Cryo-EM), and electrophoretic analysis.

Bright field and fluorescence (Figures S6B and S6C, respectively) images of the ORFTDN labelled with fluorophore FAM in the 5’-end taken on gold screen-printed electrodes (AuSPE) show that the DNA tetrahedral nanostructure has being correctly synthesized and anchored on the AuSPE thanks to the S-Au interaction between the thiol groups of the basal vertices of the ORTDN and the gold surface of the electrode.

Cryo-EM studies (Figure S6D) confirm the correct synthesis of the tetrahedral DNA nanostructure.

Figure S6E show the gel electrophoresis obtained for the single-stranded DNA sequences (Tetra A-ORF1ab SARS-CoV-2 sequence, tetra B, Tetra C, and Tetra D), the synthesized ORFTDN, and different trimer combinations lacking one strand. As it can be observed, there is a clear difference before and after the hybridization of the sequences. The ORFTDN synthesized (lane 5) moves slower than the single-stranded DNA (lanes 1 to 4) or the trimer combinations (lanes 6 to 9), due to the higher weight of the nanostructure.

**Figure S6.** ORFTDN synthesis (**A**). Bright field (**B**) and Fluorescence (**C**) optical microscopy images of a AuSPE modified with FAM labelled-ORFTDN (AuSPE/ORFTDN-FAM). Cryo-EM TDN images (**D**). Gel electrophoresis characterization (**E**) of the ORFTDN synthesis. Samples studied: lanes 1 to 4, single-stranded DNA oligonucleotides (Tetra A-ORF1ab SARSCoV-2 sequence, Tetra B, Tetra C, and Tetra D, respectively); lane 5, ORFTDN; lanes 6 to 9, trimer combinations lacking one strand (Tetra A-ORF1ab SARSCoV-2 sequence, Tetra B, Tetra C, and Tetra D, respectively).


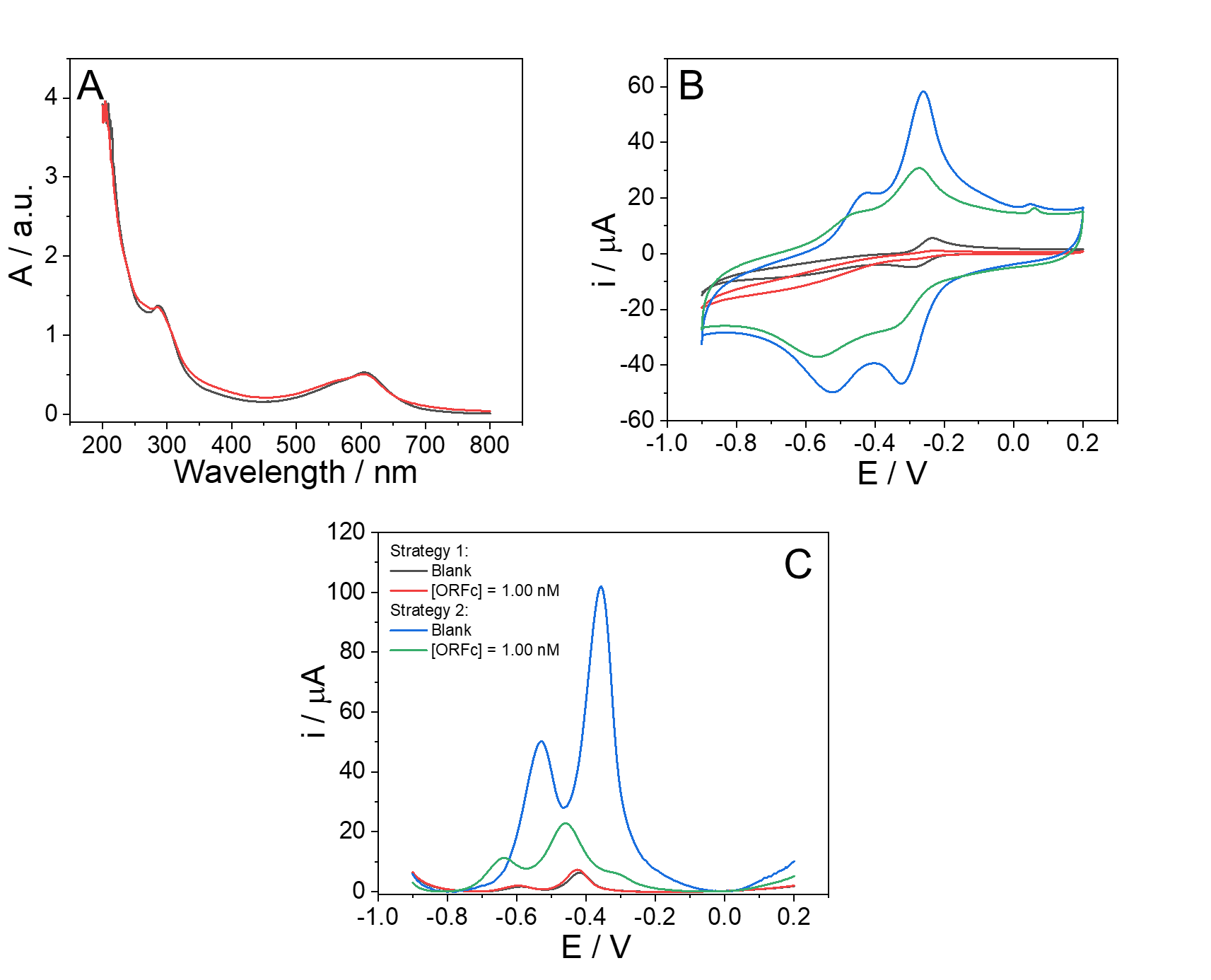


**Figure S7.** UV-Visible spectra (**A**) of two different 1:4 dilution MoS_2_-ORFTDN-CNDsTy bioconjugates in water prepared in different days. Cyclic Voltammetry (CV) (**B**) of a 1:2 solution of CNDsTy (0.280 mg·mL^-1^) in PB 0.1M pH 7.0 (black and blue lines) and of the MoS_2_-ORFTDN-CNDsTy bioconjugate (red and green lines) on a AuSPE (black and red lines) or on a CSPE (blue and green lines). Differential Pulse Voltammograms (DPV) (**C**) registered before (black and blue lines) and after (red and green lines) the hybridization of the bioconjugate with the analyte ORFc sequence, following the two different strategies of incubation and hybridization tested.

***Characterization of CSPE-MoS_2_ and CSPE-MoS_2_-ORFTDN by means of SEM, AFM and Fluorescence Microscopy***


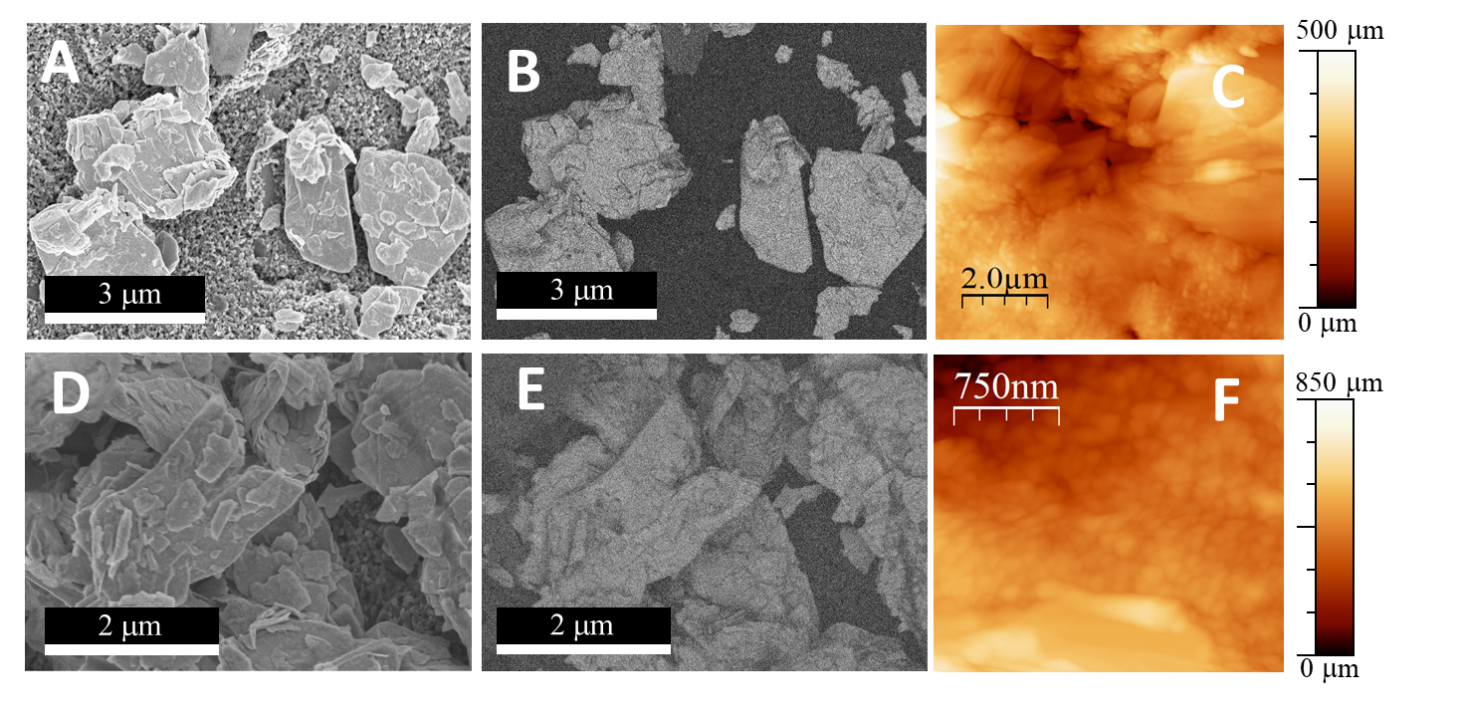


**Figure S8.** SEM secondary (**A, D**), backscattered electrons (**B,** **E**) images and AFM images (**C, F**) of CSPE- MoS_2_ (**A-C**) and CSPE-MoS_2_-ORFTDN (**D- F**).


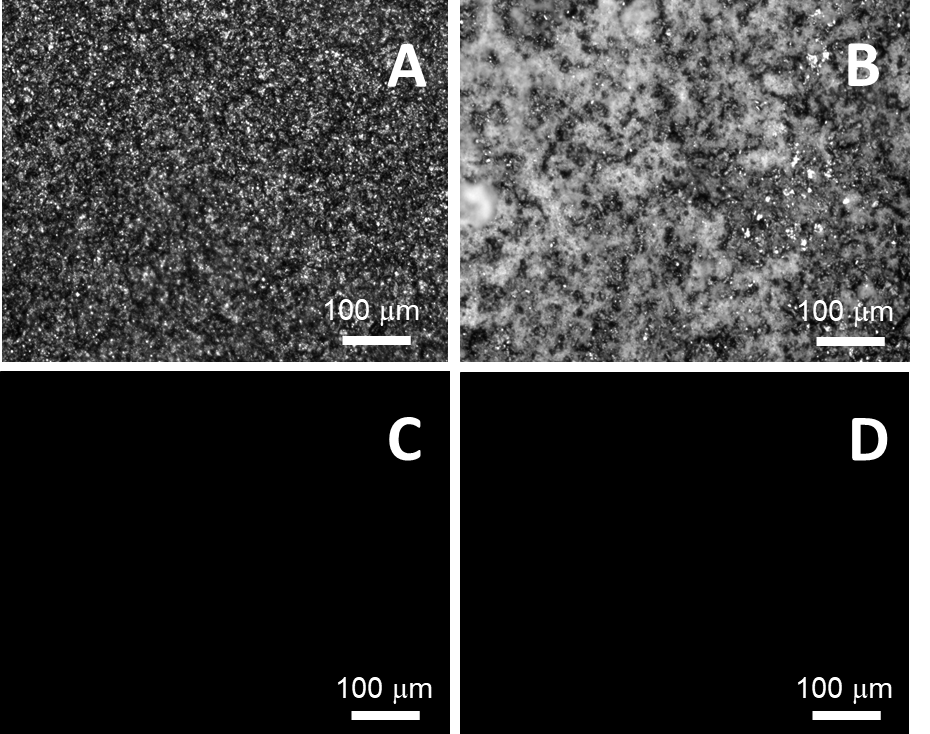


**Figure S9.** Bright field images (**A, B**) and fluorescence images (**C, D**) of a CSPE-MoS_2_ (**A**, **C**) and CSPE-MoS_2_-ORFTDN (**B**, **D**).

***Raman characterization of a CSPE modified with massive calf thymus DNA***

**Figure S10.** Raman spectra of a CSPE modified with massive calf thymus DNA (dsDNA).

***XPS characterization of a CSPE modified with molybdenum disulphide (MoS_2_)***


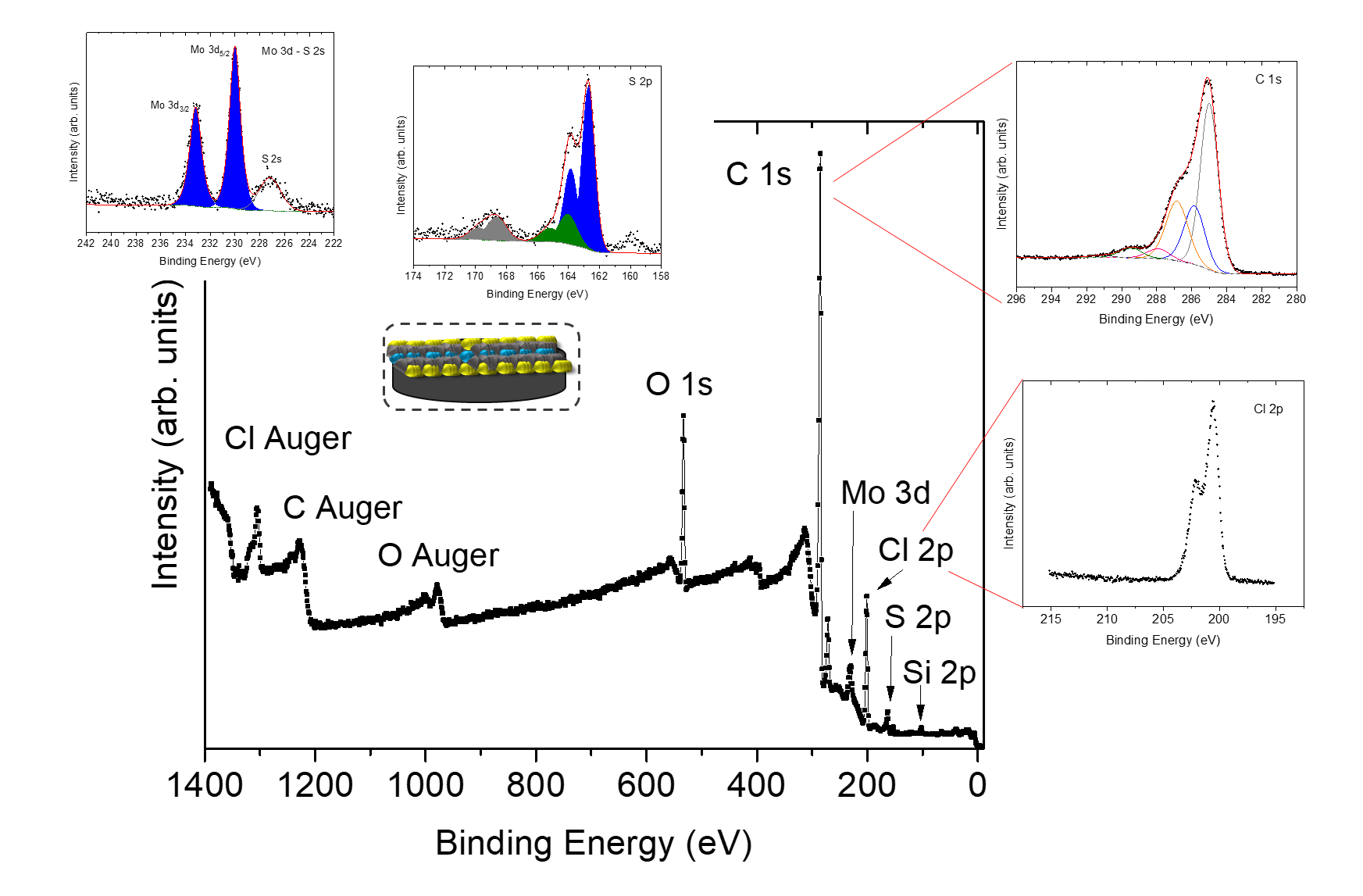


**Figure S11.** XPS core levels spectra of C 1s, Mo3d-S 2s, S 2p and Cl 2p of CSPE/MoS_2_

***XPS characterization of a CSPE modified with the ORFTDN***

***
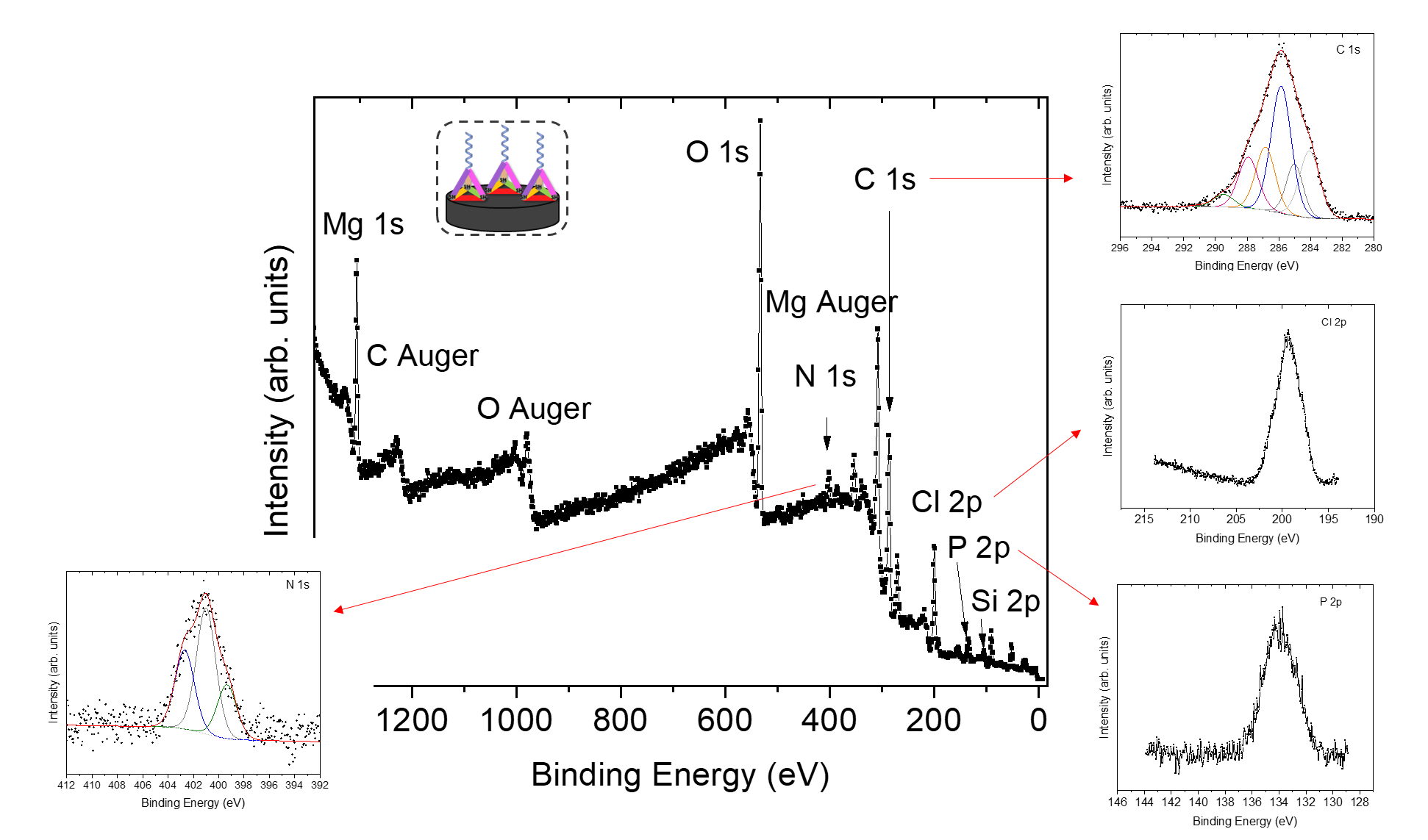
***

**Figure S12.** XPS core levels spectra of N 1s ,P 2p, C1s and Cl 2p of CSPE/ORFTDN

**

**Figure S13**. Stability study of the biosensing platform (CSPE-MoS_2_-ORFTDN-CDsTy) storage at 4 °C after the incubation with specific ORF1ab sequence at 1.00 pM concentration on different days (0 to 50 days). Data presented as mean ± standard deviation (n = 3).

**Figure S14.** Bar graph of the maximum current intensity obtained at the -0.4V characteristic redox potential of the CNDsTy for the CSPE/MoS_2_-BRCA1TDN-CNDsTy platform before (blue line) and after (red line) incubation with the specific BRA1 gen at a 1.00 pM concentration.

**References**

1. Zheng SQ, Palovcak E, Armache J-P, et al (2017) MotionCor2: anisotropic correction of beam-induced motion for improved cryo-electron microscopy. Nat Methods 14:331–332. https://doi.org/10.1038/nmeth.4193

2. Punjani A, Rubinstein JL, Fleet DJ, Brubaker MA (2017) cryoSPARC: algorithms for rapid unsupervised cryo-EM structure determination. Nat Methods 14:290–296. https://doi.org/10.1038/nmeth.4169

3. Zhang K (2016) Gctf: Real-time CTF determination and correction. Journal of Structural Biology 193:1–12. https://doi.org/10.1016/j.jsb.2015.11.003

4. Wagner T, Merino F, Stabrin M, et al (2019) SPHIRE-crYOLO is a fast and accurate fully automated particle picker for cryo-EM. Commun Biol 2:218. https://doi.org/10.1038/s42003-019-0437-z

5. Li H, Zhang Q, Yap CCR, et al (2012) From Bulk to Monolayer MoS _2_ : Evolution of Raman Scattering. Adv Funct Materials 22:1385–1390. https://doi.org/10.1002/adfm.201102111

6. Giovanelli E, Castellanos-Gomez A, Pérez EM (2017) Surfactant-Free Polar-to-Nonpolar Phase Transfer of Exfoliated MoS _2_ Two-Dimensional Colloids. ChemPlusChem 82:732–741. https://doi.org/10.1002/cplu.201700038

7. Jiang Z, Zhao C, Lin L, et al (2015) A label-free electrochemical immunosensor based on poly(thionine)–SDS nanocomposites for CA19-9 detection. Anal Methods 7:4508–4513. https://doi.org/10.1039/C5AY00576K

8. Krzyszkowska E, Walkowiak-Kulikowska J, Stienen S, Wojcik A (2017) Thionine–graphene oxide covalent hybrid and its interaction with light. Phys Chem Chem Phys 19:14412–14423. https://doi.org/10.1039/C7CP01267E
